# Characterization and risk assessment of ambient VOCs in Qingyang city, China

**DOI:** 10.1371/journal.pone.0359213

**Published:** 2026-09-28

**Authors:** Weihong Wang, Leping He, Yongqiang Wei, Fei Dou, Li Ma, Yue Zhang, Yuwei Tie

**Affiliations:** 1 Gansu Academy of Eco-environmental Sciences, Lanzhou, Gansu, China; 2 Key Laboratory of Industrial Waste Recycling and Regulation of Gansu Province, Lanzhou, Gansu, China; Nanjing University of Information Science and Technology, CHINA

## Abstract

**Introduction:**

Atmospheric volatile organic compounds (VOCs) are critical pollutants that significantly affect urban air quality and drive photochemical reaction processes. Industrial cities, especially those in western China, often face unique VOC pollution characteristics due to regional industrial structures and geographical conditions.

**Objective:**

This study aimed to characterize VOC composition, concentrations, photochemical reactivity, sources, and health risks in a western Chinese industrial city.

**Methods:**

Gaseous VOC samples were collected from four sampling sites (three urban sites and one rural site), and their compositional characteristics were analyzed in detail. To evaluate the photochemical reaction activity of the detected VOCs, the ozone formation potential (*OFP*) and propylene equivalent concentration (Propy-Equiv) method were adopted. Principal Component Analysis (PCA) was applied to identify VOC sources. Additionally, a health risk assessment was conducted to identify key pollutants posing non-carcinogenic risks.

**Results:**

The results showed that the average volume fraction of total volatile organic compounds (TVOCs) in the atmosphere of the study city was 47.94 ± 23.04 ppbv, with a total of 112 VOC species detected. Based on their molecular structures, these VOCs were classified into six categories, including 29 alkanes, 11 alkenes, 1 alkyne, 16 aromatics, 34 halogenated hydrocarbons (HHCs), and 21 oxygen-containing organic compounds (OVOCs). The volume ratio contributions of these categories to TVOCs were as follows: OVOCs (29.17%), alkanes (27.12%), HHCs (19.86%), aromatic hydrocarbons (12.33%), alkenes (10.67%), and alkynes (0.86%). Furthermore, TVOC concentrations were significantly higher in urban areas than in rural areas, and the photochemical reactivity of atmospheric components in urban areas was also higher than that in rural areas. Health risk assessment results indicated that acrolein was the primary species contributing to non-carcinogenic risks.

**Conclusion:**

Controlling alkenes and OVOCs is critical to reduce VOC pollution and photochemical risks. These findings support local air quality management.

## Introduction

In the past thirty years, volatile organic compounds (VOCs) present in the air have attracted widespread attention due to their impacts on air quality and public health [1]. VOCs have a main structure containing carbon-hydrogen covalent bonds. They enter human and animal bodies through skin contact, inhalation, or ingestion, especially those classified as hazardous air pollutants by the United States Environmental Protection Agency (EPA), which may have adverse effects on health, such as skin and eye irritation, respiratory complications, neurological damage, and visceral toxicity [1–3]. They can undergo photochemical reactions with oxidants in the atmosphere to produce ozone (O_3_) and secondary organic aerosols (SOAs), and they are also an important factor leading to atmospheric pollution [3]. Thus, VOCs exert dual impacts on atmospheric environments and human health, necessitating integrated characterization of their chemical composition, photochemical reactivity, and toxicological risks. Long-term exposure to ambient air pollution increases incidences of cardiovascular and respiratory diseases and lung cancer and shortens life expectancy [4]. Ambient air pollution, as a leading cause of global disease burden, has a greater impact in low-income and middle-income countries than in high-income countries [4–6]. Outdoor air pollution also adversely affects biodiversity, ecosystems, and ecosystem services, such as nutrient cycling [7,8]. Hence, the challenges faced in achieving future reductions in the burden of disease and in protecting public health and the global ecosystem will be enormous if pollution levels are not significantly reduced.

China’s air pollution problem has worsened due to rapid economic development and urbanization. Although government measures have led to some improvement in air quality, emissions of non-methane volatile organic compounds increased by 11% between 2010 and 2017. Air quality remains below World Health Organization (WHO) standards. Therefore, continued efforts to reduce air pollutant emissions are crucial [9–14].

VOCs are important precursors of ozone and smog in tropospheric chemistry [15]. China has experienced increasingly severe O_3_ pollution in recent years [16–22]. Research on the spatiotemporal variations in the five conventional pollutants, namely, PM_2.5_, PM_10_, SO_2_, NO_2_, and O_3_, as well as the air quality index and primary pollutants in 338 Chinese cities from 2013 to 2017, has revealed that the overall air quality has improved. However, O_3_ pollution has increased nationwide. According to the WHO Air Quality Guidelines (WHO AQG) [23], the majority of regions in mainland China have higher ozone pollution levels that exceeded the air quality recommendations. In 2023, the mean ozone concentration in 339 Chinese cities was 144 μg/m^3^, and the concentrations in the Beijing-Tianjin-Hebei (BTH) area and its surrounding regions, the Yangtze River Delta region (YRD), the Fen-Wei Plain (FWP) were 181, 158, and 167 g/m^3^ [24], respectively. Several studies have shown that VOC emissions were closely related to driving the increases in surface ozone pollution in China [3,16–22,25]. VOCs, as important precursors of ground-level O_3_ and secondary organic aerosols, have become a major challenge in China’s atmospheric environment governance and are crucial for controlling O_3_ and PM_2.5_ pollution in China [25].

A systematic review of 4593 publications on VOC emissions revealed that VOCs emitted from solvent use, transportation, the manufacturing industry, and construction, as well as fugitive emissions from fuels and non-energy products from fuels, were the subjects of most of the literature; and VOC emission characteristics, their effects on air quality and health, and VOC emissions under climate change in the past 30 years were also the main focuses of many researchers [1]. Most of the studies conducted in China mainly focused on the North China Plain (NCP), YRD, Pearl River Delta (PRD), and Sichuan Basin (SB) regions and on metropolitan areas. However, studies conducted in Northwestern China and other cities beyond metropolitan areas, which revealed that these areas are also facing serious VOC and O_3_ air pollution problems, were relatively limited [1,3,26–30]. Existing research on ambient volatile organic compounds (VOCs) across Northwest China has largely focused on large industrial metropolises represented by Lanzhou, whereas synchronous urban–rural field monitoring for small and medium-sized petrochemical cities remains extremely scarce; furthermore, most prior studies in this region have focused predominantly on VOC concentration characteristics and photochemical reactivity, with limited attention to the inhalation health risks posed by toxic VOC species to local residents.

Qingyang City is located in the eastern part of Gansu Province in northwest China and is a petroleum and natural gas chemical base in Gansu Province. The number of days on which the average ozone value (O_3_-8h) in Qingyang City exceeded 100 g/m^3^ between 2021 and 2023 was 487 d, and the number on which it was higher than 160 g/m^3^ was 25 days, with a maximum value of 194 g/m^3^ [31]. O_3_ and particulate matter (PM_10_ and PM_2.5_) were the main pollutants in Qingyang City in these three years [31]. Petrochemical enterprises are one of the important sources of VOCs, and their VOC emissions account for a large proportion of the overall industrial sources, with high activity pollutants emitted. These industrial emissions release both O_3_ precursors and toxic VOCs (e.g., benzene, 1,2-dichloroethane, and acrolein) that pose carcinogenic and non-carcinogenic risks. Thus, clarifying the photochemical reactivity and health risks of petrochemical VOCs is essential for developing comprehensive pollution control strategies in Qingyang. Nevertheless, systematic investigations that simultaneously characterize VOC profiles, spatial distributions, ozone formation potential, and inhalation health risks remain scarce for medium and small petrochemical cities across western China. Accordingly, this study performed synchronous urban-rural VOC sampling with four objectives: (1) characterize VOC concentration, composition, and spatial heterogeneity; (2) quantify photochemical reactivity via OFP and Propy-Equiv; (3) identify sources via PCA; and (4) evaluate inhalation carcinogenic and non-carcinogenic risks to identify priority pollutants. This integrated approach provides a scientific basis for coordinated VOC and O_3_ pollution control in Qingyang and similar petrochemical cities in western China.

## Materials and methods

### Study area

Based on the characteristics of the city’s functional zoning (i.e., urban, background, and industrial areas), along with field observations, representative locations were selected for analysis and testing. A total of four sampling points were set up, with sampling points 1, 2, and 3 representing the current status of the VOCs in the urban areas and sampling point 4 representing the current status of VOCs in the rural areas. Sampling point 1 (107.628°E, 35.628°N) was located upwind of the annual wind direction, and this sampling point was located at the junction between the urban and rural areas. The area surrounding the sampling point was mainly farmland, and the sampling data represent the current status of the environmental air quality in the urban-rural junction in Qingyang City. Sampling point 2 (107.625°E, 35.656°N) was located approximately 3.5 km north of sampling point 1, and reflected the current air quality status of the local petrochemical enterprises. Sampling point 3 (107.639°E, 35.708 °N) was located in the city’s central, highly populated, and commercially active district, approximately 9.5 km north of sampling point 1. Sampling point 3 reflected the current status of the ambient air quality in the city center. Sampling point 4 (geographic coordinates:107.694°E, 35.891°N) was located in a rural area, and apple orchards and corn fields surrounded this sampling point. Sampling points 1–3 were integrated to reflect the VOCs in the urban areas (S1 Fig in S1 File).

### Data and methods

#### Sampling and VOC analysis.

At the sampling points, air samples were simultaneously collected in a 3.2 L SUMMA stainless steel vacuum sampling tank fixed at a distance of not less than 1.5 meters from the ground. The sampling period was from August 8–12, 2021, with a continuous 8-hour sampling period from 9:00–17:00 every day. The sampling flow rate was 6.2 mL/min. Before sampling, the sampling tank was cleaned according to the standard procedure and evacuated to achieve vacuum (< 10 Pa). After the set 8-hour sampling period was completed, the valve was closed and sealed with a sealing cap. The entire process, encompassing sampling tank cleaning and pretreatment, field sampling operation, laboratory sample analysis and determination, as well as quality control and quality assurance (QC/QA) measures, was strictly implemented in full compliance with the national environmental protection standard of the People’s Republic of China—HJ 759–2015 *Standard Method for Determination of VOCs in Ambient Air: Sampling/Gas Chromatography-Mass Spectrometry (GC-MS)* [32,33]. Detailed information on instrument calibration, detection limits (MDLs), analytical precision (RSDs), recoveries, blank correction, and measurement uncertainty is provided in the Supplementary Information. Descriptive statistics (mean, standard deviation) were used in this study. No inferential statistical tests were performed for spatial or temporal comparisons. It should be noted that these 8-hour integrated samples provide time-weighted average concentrations representative only of daytime conditions, with no coverage of early-morning or nighttime periods.

#### Data processing method.

VOCs enter the atmosphere, and, in the presence of sunlight, undergo photochemical reactions to form O_3_ pollution, which is an important pathway for their transformation in the atmosphere. There are several methods for evaluating the photochemical reaction activity of VOCs and their contributions to O_3_ generation, such as the ozone formation potential (OFP), Propylene−Equivalent concentration (Propy−Equiv), and hydroxyl reaction rate methods [15,29,34,35]. In this study, we used the OFP and Propy−Equiv methods to conduct the analysis.

The OFP of VOC species, which is based on the maximum incremental reaction activity (*MIR*), was applied to evaluate the ozone formation [15,29,36]. The OFP is calculated as follows:


$$\begin{array}{c} OFP_{\text{i}}\;=\;MIR_{\text{i}}\;\times \;[VOCs_{\text{i}}] \end{array}$$ (1)

where *OFP*_i_ is the ozone formation potential of individual VOC species i (ppbv), and *MIR*_i_ is the maximum incremental reactivity coefficient of VOC species i. We used the MIR_i_ values used by Carter (2010) [34,37] (unit: gO_3_/ gVOCs). [*VOCs*_i_] is the concentration of individual VOC species i (ppbv).

Propy−Equiv is also commonly used to measure the activity of VOCs in photochemical reactions, and it is a measure of the concentration of species i on an OH-reactivity-based scale, with normalization to the reactivity of propylene [16,34,38].

The Prop-Equiv concentration of an individual VOC can be calculated as follows:


$$\begin{array}{c} Propy-Equiv\;(\text{i})\;=\;[VOCs_{\text{i}}]\;\times k_{\text{OH}},\text{ I }/k_{\text{OH}}{,}_{\text{C3H6}}, \end{array}$$ (2)

where *Prop-Equiv* (i) denotes a VOC compound i based on an ·OH reactivity, normalized to the reactivity of C_3_H_6_ (ppbv). k_OH, I_ is the rate constant for the reaction between species i and the ·OH radical, and k_OH,C3H6_ is the rate constant for the reaction between C_3_H_6_ and the ·OH radical (cm^3^·molecule^− 1^·s^− 1^). We used the rate constants suggested by Atkinson (1990 and 1997) [39,40] and Atkinson and Arey (2003) [41].

#### Human health risk assessment.

Toxic VOCs can enter the human body through inhalation, skin absorption, and ingestion, with inhalation being the primary pathway of exposure for environmental volatile organic compounds. Inhalation exposure is often closely associated with acute or chronic health problems, such as respiratory irritation, neurological damage, and cancer risk. Evaluating the health risks associated with environmental VOC exposure through inhalation is a commonly used assessment method [37,42,43]. The health risk assessment of VOCs was divided into a carcinogenic risk assessment and a non-carcinogenic risk assessment. The carcinogenic risk was measured using the lifetime carcinogenic risk (*LCR*, dimensionless), and the non-carcinogenic risk was measured using the hazard index (*HI*, dimensionless) [43]. Fifty-five VOC species selected based on the International Agency for Research on Cancer (IARC) and frequently reported in previous literature were calculated for the health risk assessment (S1 Table in S1 File). The *LCR*, *HI*, and adjusted exposure concentration (*EC*) were calculated as follows [43]:


$$\begin{array}{c} EC_{i}=\;\frac{C_{i}\times ET\times EF\times ED}{AT}\;\times \text{9}\text{0}\% \end{array}$$ (3)

$$\begin{array}{c} LCR=\sum_{i=\text{1}}^{n}{LCR}_{i}=\sum_{i=\text{1}}^{n}{EC}_{i}\times {IUR}_{i} \end{array}$$ (4)

$$\begin{array}{c} HI=\sum_{\text{i}=\text{1}}^{\text{n}}{HI}_{\text{i}}=\sum_{\text{i}=\text{1}}^{\text{n}}{EC}_{\text{i}}{/RfC}_{\text{i}} \end{array}$$ (5)

where *EC*_i_ is the adjusted exposure concentration of VOC species i (μg/m^3^). C_i_ is the obtained average concentration of VOC species i in the air (g/m^3^). *ET* is the air exposure time (assumed as the working time of 8 h/d). *EF* is the exposure frequency (350 days/year, assuming 15 days for annual holidays outside the area in which they live). *ED* is the exposure duration (assuming that a resident lives 24 years at the same place on average). *AT* is the average time, which was set to 70 years × 365 days/year × 24 h/day in the carcinogenic risk assessment and to *ED* × 365 days × 24 hours/day in the non-carcinogenic risk assessment. *RfC*_i_ and *IUR*_i_ are the reference concentration (μg/m^3^) and the inhalation unit risk (m^3^/g) of VOC species i, respectively (S1 Table in S1 File). For carcinogenic risk, the US EPA recommended acceptable level is 1×10^− 6^ (one in a million), and the tolerable risk is 1×10^− 4^. For non-carcinogenic risks, *HI* < 1 indicates no appreciable risk, while *HI* > 1 indicates a chance of adverse health effects occurring [43].

## Results

### Observation overview

#### Analysis of chemical composition of VOCs.

For the samples collected in this study, we analyzed the concentrations of a total of 112 VOCs, which can be classified into six categories based on the characteristics of their molecular structures: alkanes (29), alkenes (11), alkynes (one), aromatics (16), halogenated hydrocarbons (HHCs) (34), and oxygen-containing VOCs (OVOCs) (21). The alkanes, alkenes, alkynes, and aromatics were combined into the category of non-methane hydrocarbons (a total of 57) (S2 Table in S1 File). A comparison of the volume fraction ratios of the various types of VOCs at each sampling point is shown in Fig 1. The top three types of VOCs in terms of their volume ratios of the TVOCs at sampling point 1 were OVOCs, alkanes, and HHCs, accounting for 28.81%, 26.64%, and 19.15%, respectively. The top three types of VOCs at sampling point 2 were OVOCs, HHCs, and alkanes, accounting for 28.96%, 27.39%, and 26.32%, respectively. The top three types of VOCs at sampling point 3 were OVOCs, alkanes, and HHCs, accounting for 29.07%, 27.62%, and 20.07%, respectively. The top three types of VOCs at sampling point 4 were OVOCs, alkanes, and aromatics, accounting for 29.96%, 27.76%, and 26.20%, respectively. The proportion of the non-methane hydrocarbons (NMHCs) in terms of the volume fraction of the TVOCs was the highest at sampling point 4 (58.64%), followed by sampling points 1 (52.04%), 3 (50.86%), and 2 (43.65%).

**Fig 1:**
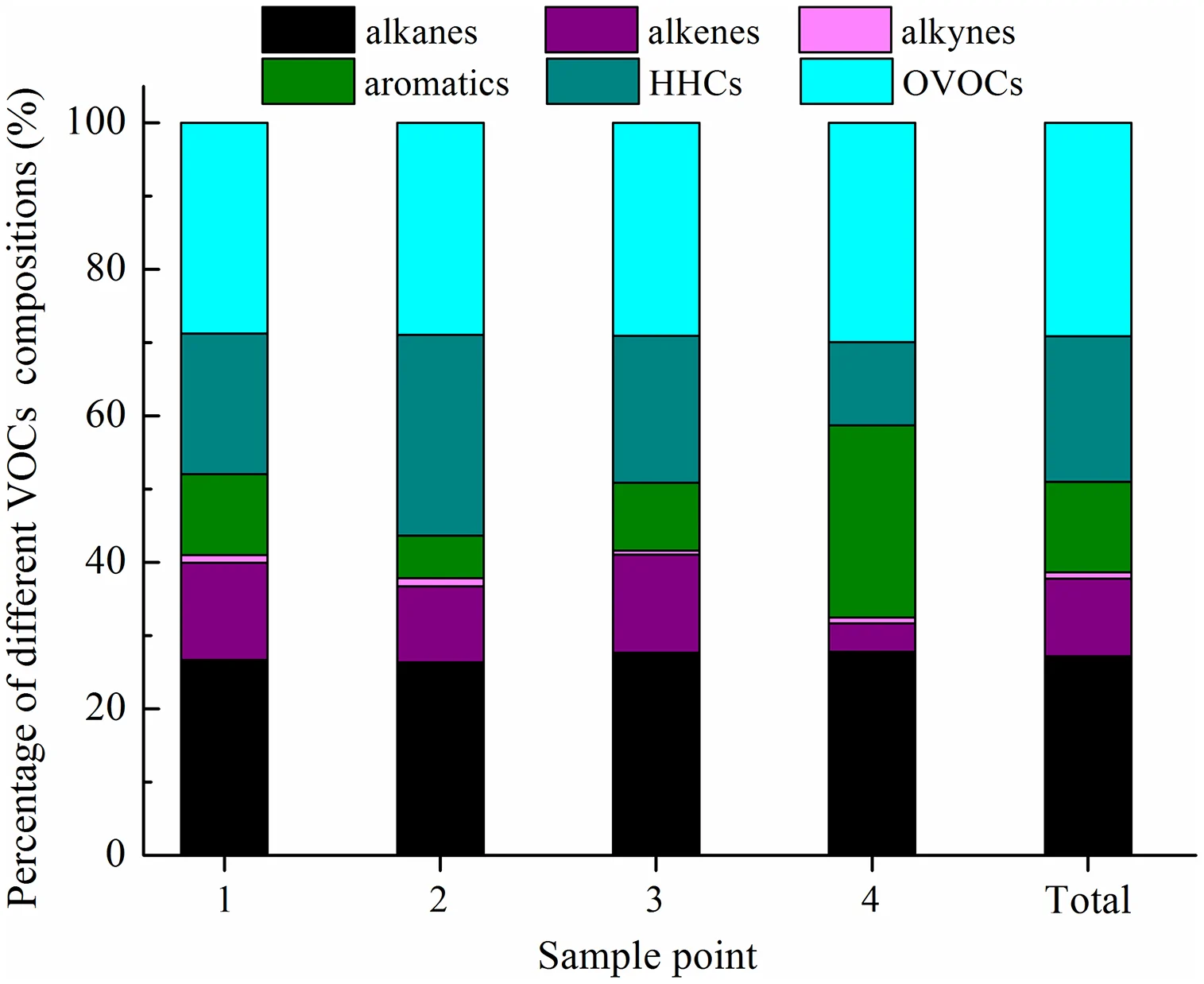
Compositions of VOCs for different species of alkanes, alkenes, acetylene, aromatics, HHCs, and OVOCs at sites 1–4. 1, the junction of the urban and rural areas; 2, urban area; 3, city center; 4, rural area; 5 Total.

Fig 2 shows the concentration characteristics of the different species of VOCs in the urban and rural air samples. The concentration of the TVOCs was higher in the urban areas than in the rural area, with average volume fractions of 50.60 ± 24.97 ppbv and 39.95 ± 15.22 ppbv, respectively. The average volume fraction of the aromatic hydrocarbons was higher in the rural area than in the urban areas. Those of the other species of VOCs were lower in the rural area than in the urban areas. The order of the average volume fractions of the different types of VOCs in the urban areas was OVOCs (28.96%)> alkanes (26.95%)> HHCs (22.09%)> alkenes (12.45%)> aromatic hydrocarbons (8.68%)> alkynes (0.87%). In the rural area, the order of the VOCs was OVOCs (29.96%)> alkanes (27.76%)> aromatic hydrocarbons (26.20%)> HHCs (11.36%)> alkenes (3.9%)> alkynes (0.82%). In the urban areas, the sum of the proportions of the OVOCs, alkanes, and HHCs was 78%. In the rural area, the sum of the proportions of the OVOCs, alkanes, and aromatic hydrocarbons was 84%.

**Fig 2:**
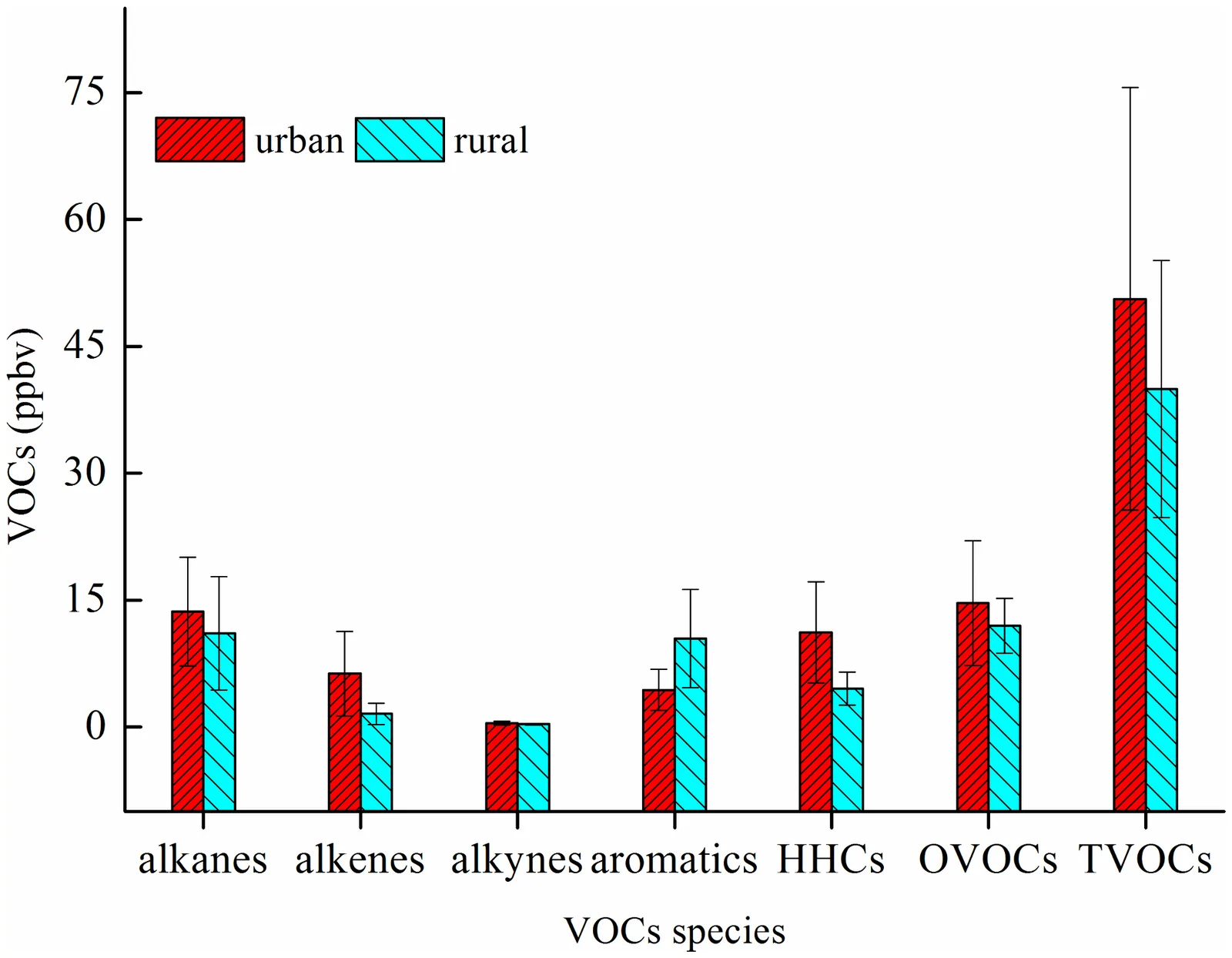
The concentrations and compositions of the different species of VOCs in the urban and rural areas.

The overall VOC concentration characteristics in Qingyang City during the comprehensive analysis observation period are analyzed and the results showed that the average volume fraction of the TVOCs at the monitoring points was 47.94 ± 23.04 ppbv, and the volume fraction of the OVOCs was highest, with an average value of 13.98 ± 6.62 ppbv, followed by the alkanes, HHCs, aromatic hydrocarbons, olefins, and alkynes, with average values of 13.00 ± 6.40 ppbv, 9.52 ± 5.99 ppbv, 5.91 ± 4.34 ppbv, 5.11 ± 4.84 ppbv, and 0.41 ± 0.20 ppbv, respectively. The average value of the NMHCs was 24.44 ± 12.17 ppbv. The volume ratios of each type of VOC to the TVOCs in descending order was as follows: OVOCs, alkanes, HHCs, aromatics, alkenes, and alkynes, with proportions of 29.17%, 27.12%, 19.86%, 12.33%, 10.67%, and 0.86%, respectively (Fig 1). The average volume fraction of the NMHCs was 24.44 ± 12.17 ppbv, accounting for 50.97% of the TVOCs. It was observed that the OVOCs, alkanes, and HHCs were the main VOC components in the atmosphere of Qingyang City.

#### Analysis of volume fractions of main VOCs.

Fig 3 shows the top 10 VOC species by volume fraction in the urban and rural areas during the observation period. The top 10 VOC species in the urban areas were methyl chloride, n-butane, iso-pentane, methylacrolein, dichloromethane, 1-butene, crotonaldehyde, benzene, acetaldehyde, and isobutane, with a combined volume fraction of 15.53 ppbv, accounting for 30% of the TVOC volume fraction in the urban areas. These 10 VOC species included three alkanes, one alkene, one aromatic hydrocarbon, two HHCs, and three OVOCs.

**Fig 3:**
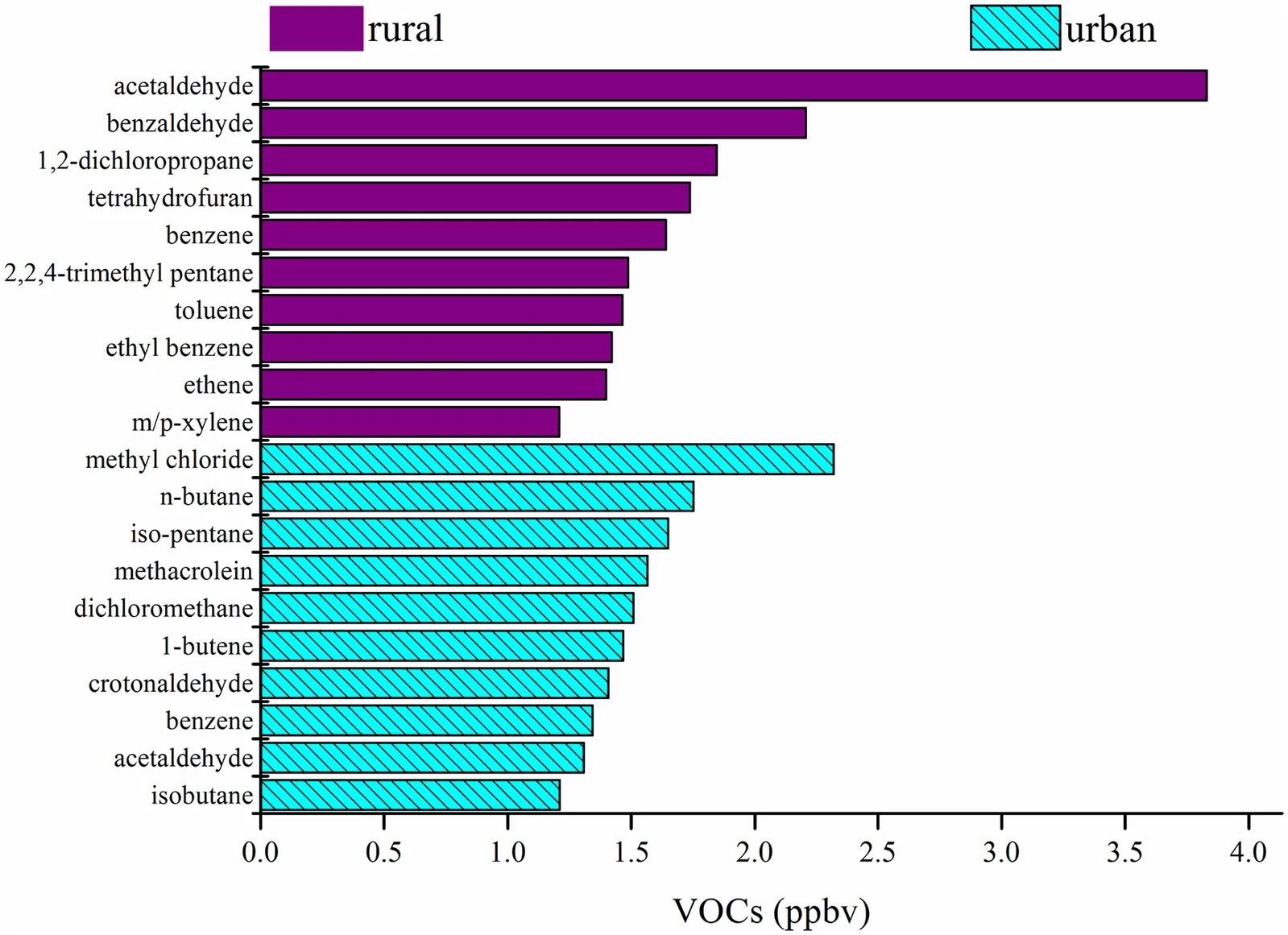
The top 10 VOC species in the volume fraction in the urban and rural areas.

The top 10 VOC species in the rural area were acetaldehyde, benzaldehyde, 1,2-dichloropropane, tetrahydrofuran, benzene, 2,2,4-trimethylpentane, toluene, ethylbenzene, ethylene, and m/p-xylene, with a combined volume fraction of 18.24 ppbv, accounting for 45.66% of the TVOC volume fraction in the rural area. These 10 VOC species included one alkane, one alkene, four aromatic hydrocarbons, one HHC, and three OVOCs.

The top 10 species in terms of the VOC volume fraction among the monitoring points in Qingyang City were acetaldehyde, chloromethane, benzene, n-butane, isopentane, methacrylaldehyde, dichloromethane, butenal, 1-butene, and toluene. The combined volume fraction of these substances (13.35 ppbv) accounted for 27.8% of the total VOC volume fraction (47.94 ppbv) in the entire region.

#### Chemical reactivity of VOCs.

The OFPs of the volume fractions of the alkanes, alkenes, alkynes, aromatics, HHCs, and OVOCs in the urban and rural areas were calculated. The contributions of these different types of compounds are shown in Fig 4. The average OFP was higher in the urban areas than in the rural area, with values of 176.45 ppbv and 131.45 ppbv, respectively. Alkenes and OVOCs were the largest contributors to urban OFP, with values of 68.08 ppbv (38.58%) and 66.23 ppbv (37.53%), respectively. Next were the aromatic hydrocarbons and alkanes, with OFP values contributing 11% and 9.6%, respectively. The contributions of the HHCs and alkynes to the OFP value were both less than 5%. Aromatic hydrocarbons and OVOCs were the largest contributors to rural OFP, with OFP magnitudes of 58.46 ppbv and 45.60 ppbv, collectively accounting for 79.16% of total rural OFP. Next were the alkenes and alkanes, and the contributions of the OFP values of the HHCs and alkynes were less than 2%. The OFP value of the NMHCs in the urban areas was 104.92 ppbv, which was higher than that in the rural area (84.08 ppbv).

**Fig 4:**
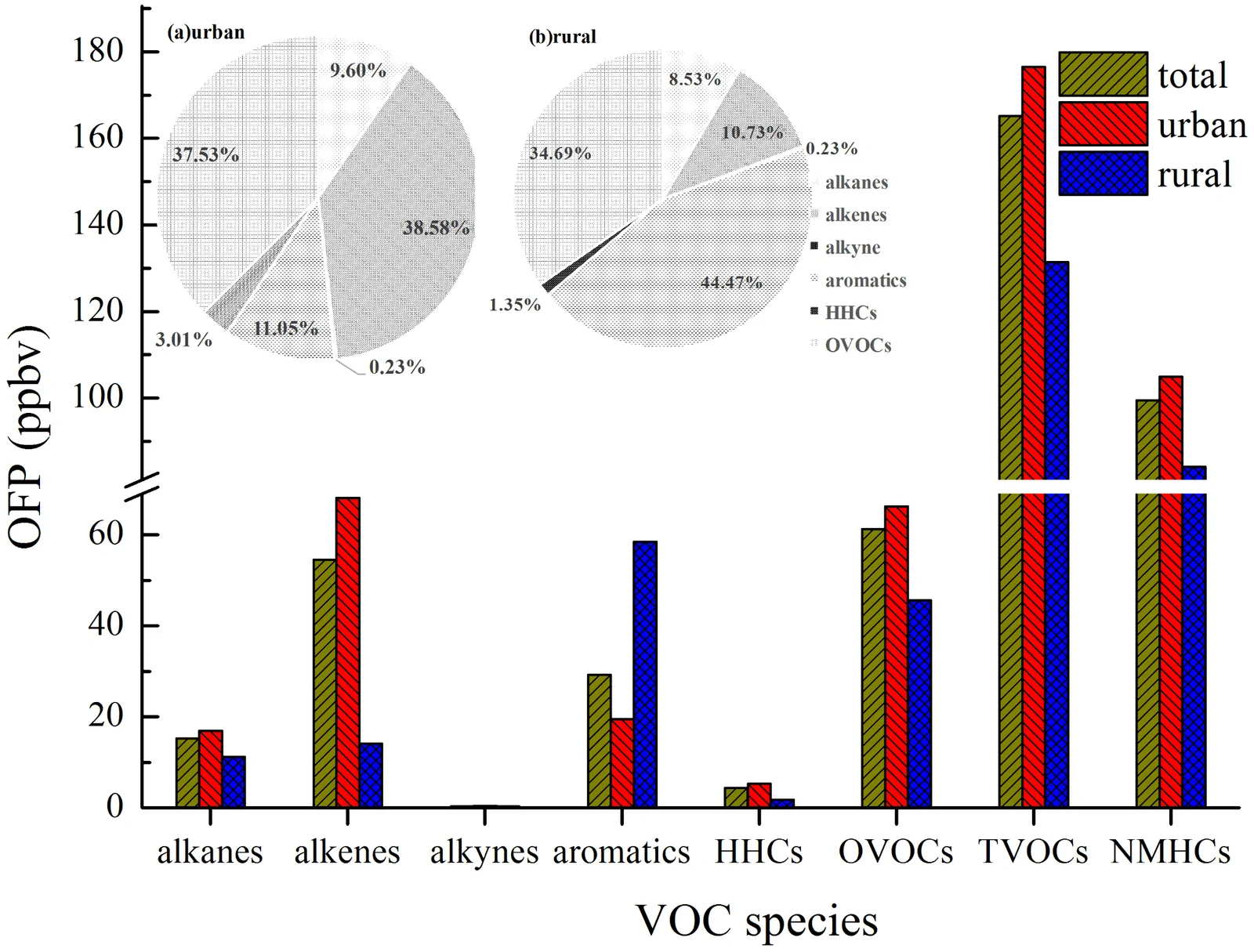
OFP values and compositions of the different species of alkanes, alkenes, acetylene, aromatics, HHCs, and OVOCs in the (a) urban and (b) rural areas.

Overall, the TVOCs corresponded to an OFP value of 165.19 ppbv in Qingyang City. The OFP values of OVOCs, alkenes, and aromatics were 61.28, 54.59, and 29.23, respectively, while those of the other VOC species were lower than 20 ppbv. The contributions of different VOC species to the total OFP were as follows: OVOCs led with 37.10%, followed by alkenes at 33.05%, aromatic hydrocarbons at 17.69%, alkanes at 9.26%, HHCs at 2.67%, and alkynes with the lowest contribution at 0.23%. The contribution of the NMHCs to the OFP was 60.24%.

Fig 5 shows the top 10 VOC species that contributed to the OFP values in the urban and rural areas. The top 10 species in the urban areas were 1-butene, trans-2-butene, crotonaldehyde, methacrolein, cis-2-butene, 1,3-butadiene, acetaldehyde, propionaldehyde, trans-2-pentene, and butanal, with a collective OFP value of 94.30 ppbv, accounting for 53.44% of the total OFP value in the urban areas. The top 10 species contributing to the OFP in the rural area were acetaldehyde, m/p-xylene, ethene, tetrahydrofuran, o-xylene, toluene, 1,3,5-trimethylbenzene, ethylbenzene, methyl methacrylate, and 1,2,3-trimethylbenzene, with a collective OFP value of 90.66 ppbv, accounting for 67.43% of the total OFP value in the rural area. Overall, the top 10 species in Qingyang City were acetaldehyde, 1-butene, crotonaldehyde, trans-2-butene, m/p-xylene, methacrolein, 1,3-butadiene, cis-2-butene, ethene, and propionaldehyde, with a collective OFP value of 82.16 ppbv, accounting for 49.7% of the total OFP value. By comparing the top 10 species in terms of the VOC volume fraction (Fig 3) with the top 10 species in terms of the OFP (Fig 5), we found that the species with higher VOC volume fractions did not necessarily have higher OFP values. For example, in the urban areas, four species ranked among the top 10 in both volume fraction and OFP value, namely, 1-butene, methacrylaldehyde, butenal, and acetaldehyde. Among them, 1-butene ranked sixth in terms of volume fraction but first in terms of OFP value.

**Fig 5:**
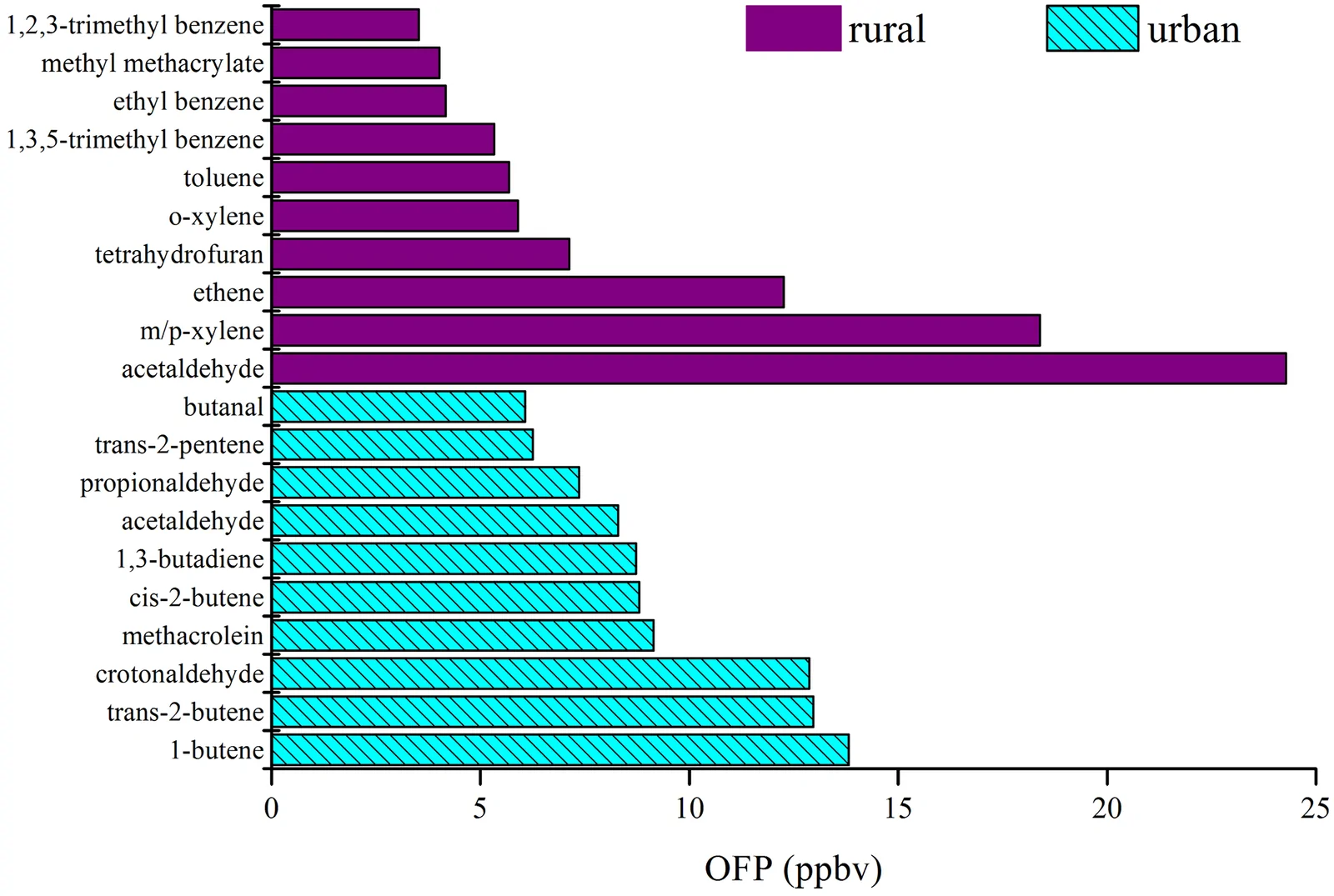
OFP values of the top 10 species during the observation period.

The Propy-Equiv values of the different species of VOCs were calculated (S2 Fig in S1 File). The average Propy-Equiv values were higher in the urban areas than in the rural area, with values of 23.67 ppbv and 15.32 ppbv, respectively. Alkenes and OVOCs were the largest contributors to urban Propy-Equiv values, collectively accounting for 79.61% of the total urban Propy-Equiv. Aromatic hydrocarbons and OVOCs dominated the rural Prop-Equiv budget, together contributing 76.83% to the total calculated Prop-Equiv. In Qingyang City as a whole, the Prop-Equiv value of the TVOCs was 21.59 ppbv, and the combined contribution of the alkenes and OVOCs to the Prop-Equiv of the TVOCs was 71.14%.

### Principal component analysis (PCA)

Principal Component Analysis (PCA) was employed for dimensionality reduction of atmospheric pollutant concentration data at each sampling site, and Factor Analysis (FA) was applied for source apportionment. All data were standardized using the Z-score method, and the Varimax orthogonal rotation was adopted. The results are presented in S3 Table in S1 File.

PCA results showed that four principal components were extracted with the criterion of eigenvalue > 1, and the cumulative variance contribution rate reached 92.08%, indicating that the first four principal components could sufficiently represent the overall information of 112 atmospheric pollutants, with an excellent dimensionality reduction effect. The first principal component (PC1) exhibited the highest variance contribution rate of 38.00%, serving as the dominant factor affecting the composition of atmospheric pollutants. The second, third, and fourth principal components accounted for 24.59%, 18.36%, and 11.13%, respectively, which together constituted the main sources of variation in pollutants. The rotated loading matrix (S4 Table in S1 File) demonstrated that PC1 was dominated by halogenated alkanes, with high-loading pollutants including 1,2-dichloropropane and hexachloro-1,3-butadiene, representing industrial emissions and solvent volatilization. PC2 was mainly controlled by esters, ketones and aromatic hydrocarbons, such as methyl methacrylate, ethyl acetate, and m-ethyltoluene, reflecting the characteristics of urban–rural domestic sources and solvent usage. PC3 was closely related to aldehydes and halogenated aromatics, represented by benzaldehyde and monochlorobenzene, indicating domestic emissions and partial industrial sources. PC4 primarily represented the variation of halogenated alkenes, including cis-1,3-dichloropropene and o-dichlorobenzene, reflecting the impacts of specific industrial and traffic emissions.

Overall, VOC pollution in the study area was characterized by multi-source compounding and significant urban–rural differences. PCA effectively identified VOC associations and potential sources, providing a scientific basis for atmospheric VOC source apportionment and control.

### Health risk assessment

According to the LCR and HI calculation methods, the health risks of exposure to toxic and harmful volatile organic compounds in the urban, rural, and overall areas were evaluated (Fig 6 and S3 in S1 File). The LCR values of the VOC substances varied from 7.25 × 10^− 11^ to 2.23 × 10^− 6^, which were all below tolerable levels. The LCR of the overall area was 8.48 × 10^− 6^, with values of 9.06 × 10^− 6^ in the urban areas and 6.75 × 10^− 6^ in the rural area. The LCR value was slightly higher in the urban areas than in the rural area. Overall, no significant potential health risks were identified in the city.

**Fig 6:**
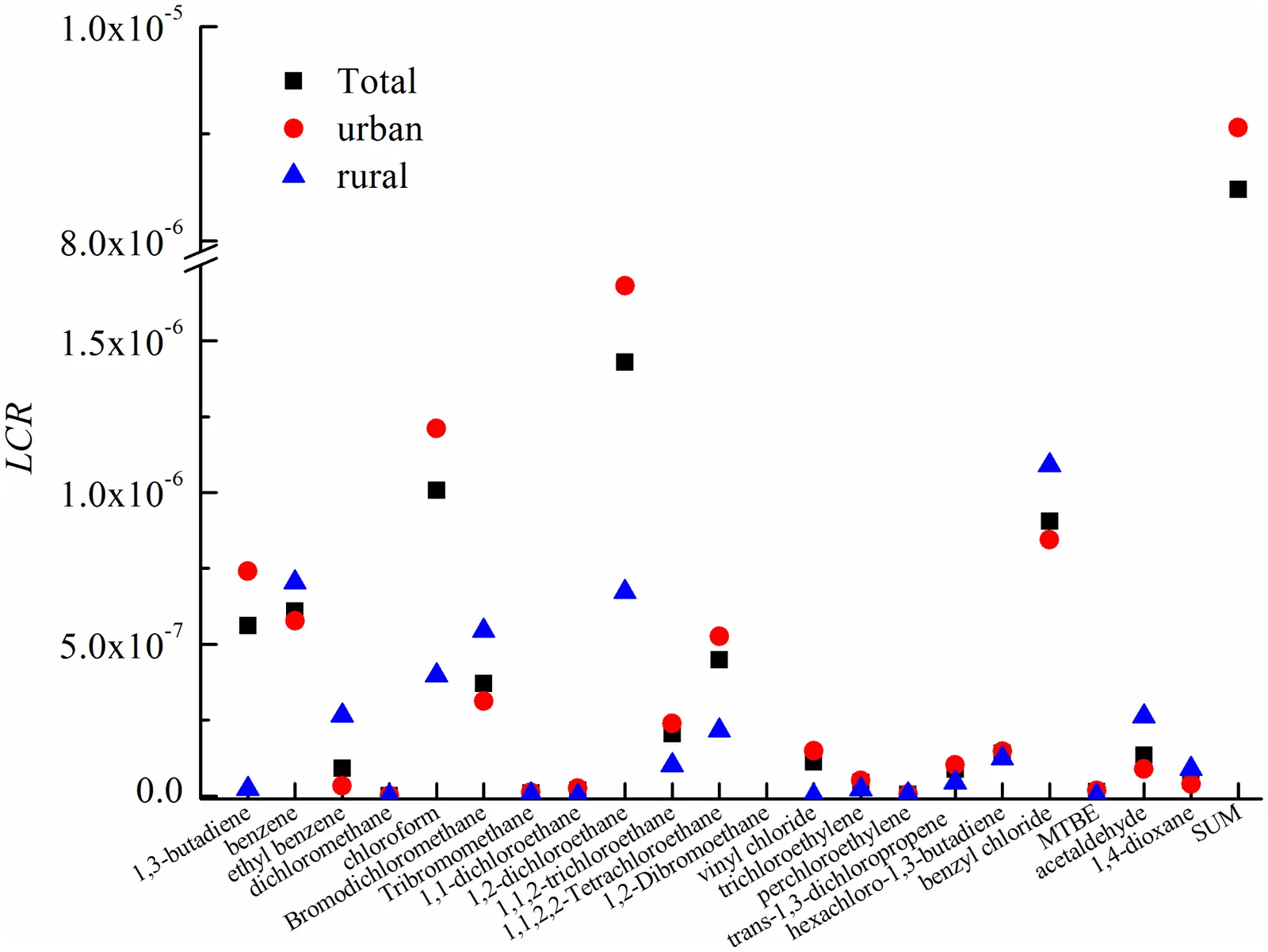
Carcinogenic risks of hazardous VOC species.

For the non-carcinogenic risk assessment, in the urban areas, only acrolein had an HI_i_ value of greater than 1, was, with a value of 3.7, while none of the substances had values of greater than 1 in the rural areas. The total HI_i_ value of the VOCs in urban areas was 4.1, which was higher than that in the rural area (1.1). In the overall area, the HI_i_ value of acrolein was 2.9, and the total HI value of the VOCs was 3.4.

## Discussion

### Concentration levels, composition, and spatial heterogeneity

Currently, the VOC species monitored in published papers (108) mainly include alkanes, olefins, aromatics, OVOC, HHCs, sulfides, and nitrogen-containing VOCs (NVOCs), and the number of species monitored in published papers ranged from five to 119 between 2015–2021 [3]. Among the total publications, about 34% monitored 50–60 VOCs, while 26 studies (approximately 24% of the total) monitored 95–105 VOCs. Overall, less than 100 VOC species were monitored in approximately 79% of the studies. Based on the frequency of VOC occurrence in the published literature, Yang et al. listed the top 100 monitored VOCs, including 29 alkanes, 13 alkenes, 16 aromatic hydrocarbons, one alkyne, 21 HHCs, 19 OVOCs, and one NVOC [3]. Compared with the review [3], our study did not detect sulfides or NVOCs in the city’s atmosphere. Of the 112 VOCs species detected, all were listed in their review, except for benzaldehyde.

Liu et al. [44] found that the TVOC concentrations in Northern and Southern China were 47.4 ± 38.0 and 41.3 ± 17.3 ppbv, respectively. The VOC concentrations at the background sites were in the range of 4.78–12.17 ppbv, and the mean value was 12.5 ± 6.7 ppbv. Relatively speaking, the TVOC concentration in Qingyang City was 3.8 times higher than that at the background sites and is on par with the VOC concentration in Northern China. On the Fenhe Plain in Northern China, which is a typical coal resource-based region, the ranges of the TVOC concentrations in three cities (Lvliang, Linfen, and Yuncheng) were 44.4 ± 24.9 to 37.5 ± 25.0 ppbv, and the proportions of the VOC groups from high to low were alkanes (43.5–31.5%), OVOCs (28.9–24.3%), alkenes (20.1–11.8%), aromatics(10.2–6.3%), HHCs (7.3–5.7%), alkynes (8.1–4.0%), and carbon disulfide (0.5–0.3%) [44].

Changzhou is one of the cities with the most serious O_3_ pollution in the central Yangtze River Delta region in China. The average concentration of VOCs was 39.52 ± 23.14 ppb. Ninety-nine VOC species were detected, including 28 alkanes, 11 alkenes, acetylene, 17 aromatics, 33 halocarbons, seven OVOCs, and two other VOCs. Alkanes (39.4%), OVOCs (23.1%), and halocarbons (16.1%) were the major contributors to the total VOC concentration, with a total relative contribution of 74.2% [45]. Wang et al. [46] found that the total VOC mixing ratios ranged from 27.10 ± 3.35 to 39.09 ± 10.84 ppb in Wuhu City, where OVOCs, alkanes, and halocarbose were the dominant groups, together accounting for 75.89–82.24% of TVOCs. Zeng et al. [33] detected and analyzed 115 VOC species, including 21 OVOCs, in urban Zhengzhou area in central China. The average concentration of the TVOCs was 162.25 ± 71.42 μg/m^3^, and OVOCs made the largest contribution (34.49%), followed by alkanes (24.29%), aromatics (19.49%), HHCs (14.81%), alkenes (4.38%), CS_2_ (1.59%), and alkynes (0.95%).

Alkanes were the dominant VOC group in the North China Plain, Yangtze River Delta, and Pearl River Delta regions and are associated with fuel combustion [44,47]. In most large and medium-sized urban areas, such as Beijing, Shanghai, and Wuhan, alkanes account for the largest proportion of VOCs (36–56%) due to the high demand for fossil fuels and their relatively long atmospheric residence times [33]. Cui et al. [48] reported an average VOC concentration of 34.9 ppbv in Beijing, with OVOCs, alkanes and halocarbons dominating total VOCs. The VOC characteristics in Qingyang City were similar to those reported for Beijing [48].

The sources of OVOCs are complex and include anthropogenic sources (such as vehicle exhaust, biomass combustion, and industrial solvents), biogenic sources, and secondary transformations [44,49]. OVOCs are the main components of VOCs in petrochemical areas [50], among which aldehydes and ketones are related to secondary reactions in the atmosphere [51]. Shen et al. [52] analyzed VOCs from various petrochemical processes, including oil refining, storage tanks, and wastewater treatment, and found high levels of OVOCs in the delayed coking process. Specifically, MTBE, methyl ethyl ketone, and acrolein were detected, with MTBE at 23 μg/m^3^ and the others at extremely low levels. MTBE was also prominent in gasoline and diesel vapors, ranking among the top 10 species. In Qingyang City, these three substances were also detected, and their average volume fractions were obviously higher in the urban areas than in the rural area. Twenty-one OVOC compounds were detected in the city. Among them, acetaldehyde, crotonaldehyde, and methacrolein were the top three in the urban areas, and tetrahydrofuran, benzaldehyde, and acetaldehyde were the top three in the rural area. C4–C6 alkanes, propylene, C6–C8 aromatics, and MTBE were the major species emitted from the petrochemical industry in Wuhan City. n-Hexane and iso-pentane were the most frequently monitored species in different process units and accounted for a large proportion of the total VOC emissions in the petrochemical industry [52]. Alkanes, aromatic compounds, and OVOCs that were detected in Wuhan City were all detected in Qingyang City. In this study, the average volume fractions of n-hexane and iso-pentane were obviously higher in the urban areas than in the rural area.

According to the VOCs emission characteristics of the eight main primary source categories in China, the sources of the alkane emissions, which accounted for more than 25% of the TVOCs, were mainly from on-road mobile, industrial processes, and fuel oil storage and transportation sources. In particular, alkanes from fuel oil storage and transportation sources were major contributors, accounting for 73% [49]. Compared with Qingyang City, the chemical composition of the alkanes analyzed from the petroleum refinery in Wuhan did not include n-dodecane. This substance did occur in the atmosphere of Qingyang City, and its concentration was lower in the urban areas than that in the rural area. The on-road mobile source was one of major VOC contributors in most regions in China. It has been found that the chemical compositions of VOCs from gasoline-powered vehicles were very similar to those from diesel vehicles for compounds containing < 10 carbon atoms. Ethylene, isopentane, benzene, and toluene were the main species in exhaust emissions [50]. Compared to gasoline vehicles, exhaust from diesel vehicles was responsible for significant emissions of species such as n-nonane, n-decane n-undecane, and n-dodecane [49 50 52]. 2-methylpentane and 1,3-butadiene can be used as tracers for vehicle exhaust [50], and in Qingyang City, the levels of both were higher in the urban areas than in the rural area. The presence of n-undecane and long-chain hydrocarbons such as nonane, n-decane, and undecane is also associated with asphalt emissions, which come from sources such as heavy oil processing, road construction, and pavement repair [43]. In Qingyang City, the levels of the heavy alkanes were higher in the rural area than in the urban areas, while the levels of most of the light alkanes were lower in the rural area than in the urban areas, indicating that the sources of the air pollution in the rural area were closely related to diesel vehicle exhaust emissions and asphalt emissions.

HHCs were the main components of the VOCs in the atmosphere of Qingyang City. HHC species have been found in stationary combustion, solvent use, industrial process, and biomass burning sources [49]. In the oil refinery sector, HHC species (e.g., Freon114(C_2_F_4_Cl_2_), chloromethane, dichloromethane, chloroform, carbon tetrachloroide, 1,2-dichloroethane, and 1,1,2-trichloroethane, which had concentrations of 1–3 μg/m^3^) have mainly been detected in regular and reduced pressure distillation, reforming hydrogenation, catalytic hydrogenation with wax oil, and delayed coking process sources [52]. A total of 24 HHC compounds have been detected in automobile manufacturing enterprises, printed circuit boards manufacturing enterprises, and gas station emissions sources. Among these substances, those with concentrations of greater than 50 μg/m^3^ included Freon11(CFCl_3_), chloromethane, dichloromethane, 1,2-dichloroethane, and 1,1,2-trichloroethane [52]. Among these 24 HHCs, all except CCl_4_ were detected in Qingyang city. A total of 35 HHC compounds were detected in the fine chemical industrial park in Kaifeng (FCIPK), Henan Province, China. The average concentration of TVOCs in the FCIPK was 320.62 μg/m^3^ (85.00 ppb). Aromatics (37.72%) and halohydrocarbons (31.33%) were the major components, followed by OVOCs (16.83%), alkanes (10.95%), alkenes (2.43%), CS_2_ (0.53%), and alkynes (0.23%). Toluene, 1,2-dichloroethane, and dichloromethane were the most abundant species, with average concentrations of 56.37 μg/m^3^, 43.83 g/m^3^, and 25.26 g/m^3^, respectively [37]. Compared with the FCIPK, the 34 types of HHC in Qingyang City were the same as those in the industrial park, except for CCl_4_, which was not detected. Ethylene dichloride, vinyl chloride, ethyl alcohol, and acetone were the predominant ambient VOC species detected around a petrochemical complex and an oil refinery in Izmir, Turkey [50]. In this study, vinyl chloride, 1,1-dichloroethene, cis-1,2-dichloroethene, and acetone were all detected, with acetone having the highest level (0.86 ± 0.46 ppbv).

The characteristics of ambient VOCs in urban Beijing revealed that alkanes were the most abundant major group during the sampling period, accounting for more than 30%, followed by OVOCs (20.3–27.9%), HHCs (15.8–24.3%), aromatics (12.0–13.3%), alkenes (4.7–11.2%), alkynes (2.3–5.3%), and other VOCs (0.4–0.7%). There were 28 halohydrocarbons compounds, and chloromethane (5.580 μg/m^3^) had the highest concentration [43]. In Qingyang, the levels of methyl chloride, dichloromethane, and 1,2-dichloroethane, as well as the total level of the HHCs, were higher in the urban areas than in the rural area, indicating that there was a close relationship between the urban atmospheric environment and industrial production.

The sources of the aromatic hydrocarbons include almost all of the Level-I source categories, except for cooking. The proportion of aromatic hydrocarbons generated from fixed combustion, industrial processes, solvent usage, and biomass combustion is higher than those of the other sources; among these sources, benzene and toluene are both among the top ten contributors to aromatic hydrocarbons [49]. In Qingyang, these two substances were also among the top two aromatic hydrocarbons. In Beijing, 11 types of alkenes and acetylene were detected [43]. In this study, we detected these same 11 alkene compounds and acetylene, accounting for 10.49% and 0.84% of the TVOCs. Alkenes mainly come from road and non-road mobile sources [49].

In Qingyang, the acetaldehyde content was higher in the rural area than in the urban areas. Acetaldehyde was also ranked first, and n-butane, methylene chloride and toluene were also among the top ten most abundant species of VOCs in terms of concentration in Beijing [43]. Acetaldehyde is mainly from mobile, biomass burning, and cooking sources, is considered to be a hazardous air pollutant by the U.S. EPA, plays an important role in O_3_ production, and is a highly abundant OVOC in many regions [33 49]. Biofuel is still widely used for cooking and heating in rural China. Hence, biomass burning is one of the important sources of acetaldehyde [43].

The benzene/toluene ratio (B/T) provides additional insight into source characteristics. In Qingyang, the average B/T ratio (>1) is higher than the range typical of vehicle-dominated emissions (0.4–0.8) and instead indicates a stronger influence from coal combustion or biofuel burning [43]. It has been reported that the high proportion of methacrolein was associated with the typical oxidation products of isoprene at urban sites in Beijing [43]. Similarly, in this study, we found that the methacrolein level was higher in the urban areas than in the rural area. In the Level-I source categories, the top two species of compounds emitted from fuel oil storage and transportation are n-butane and isopentane [49].

### OFP and reactivity-driven control implications

In Qingyang, the TVOCs corresponded to an OFP value of 165.19 ppbv, and the VOC species contributions to the total OFP were as follows: OVOCs (37.10%)> alkenes (33.05%)> aromatic hydrocarbons (17.69%)> alkanes (9.26%)> HHCs (2.67%)> alkynes (0.23%). The combined contributions of OVOCs, alkenes, and aromatics to the total OFP amounted to 87.8%. Alkenes contain reactive carbon-carbon double bonds that readily react with OH radicals under solar irradiation to generate peroxy radicals, which drive the photochemical ozone-formation cycle. Aromatic hydrocarbons can also produce peroxy radicals via OH-initiated oxidation, although their reactivity differs substantially across substituted-benzene species. Many OVOCs, particularly aldehydes, undergo direct photolysis and act as radical reservoirs, further accelerating ozone production. In contrast, saturated alkanes and halogenated HHCs possess strong, stable chemical bonds; their slow oxidation kinetics yield low MIR values, so they make minor contributions to ozone formation even at high ambient concentrations. This concentration-reactivity decoupling explains why OVOCs, alkenes, and aromatics dominate OFP, despite not always representing the largest mass fraction of ambient VOCs [34,39–41].

In Beijing, the TVOCs corresponded to an OFP value of 307.3 ± 226.8 μg/m^3^, and the contributions of the VOC groups to the OFP were as follows: OVOCs (31.0%)> alkenes (30.4%)> aromatics (28.0%)> alkanes (8.9%)> alkynes (1.4%)> HHCs (0.4%). The concentrations of the alkanes and HHCs were high, but their chemical reactivities were low, so their contributions to the OFP were much lower than those of the OVOCs, aromatics, and alkenes [43]. In Wuhu City, the OFP results showed that alkenes, aromatics, and OVOCs were the key components involved in O_3_ formations, contributing 27.6–39.5%, 22.0–32.0%, and 16.9–27.8% of the total calculated OFP, respectively [46]. In urban Zhengzhou, the average OFP of the TVOCs was 478.39 ± 228.50 μg/m^3^, which was dominated by OVOCs (41.77%), aromatics (33.82%), and alkenes (14.72%), followed by alkanes (8.64%), HHCs (0.60%), alkyne (0.31%), and CS_2_ (0.13%); acetaldehyde had the highest OFP value, and the OVOCs, especially aldehyde substances, also played an important role in O_3_ generation [33]. This research indicates that OVOC, aromatic hydrocarbons, and alkenes play important roles in ozone formation in the lower atmosphere.

There was a significant difference between the compounds with higher mass concentrations in the atmosphere in Qingyang City and the chemicals that contribute significantly to ozone formation. Therefore, in order to improve the atmospheric environmental quality in the urban areas, separate control lists should be established for emission concentrations and atmospheric reactivity in order to carry out emission reduction work more scientifically. As mentioned above, the alkenes and OVOCs mainly came from petrochemical enterprises and motor vehicle exhaust. Therefore, from the perspective of controlling the O_3_ concentration in the lower atmosphere, Qingyang City should focus on the supervision of petrochemical enterprises and motor vehicles to reduce VOC emissions.

The chemical reaction activity patterns of the various types of VOCs determined using the Propy-Equiv and OFP methods suggest that the alkenes and OVOCs were the main factors affecting the O_3_ concentration in the urban areas of Qingyang. The chemical reactivities of the VOC species varied greatly and contributed differently to the O_3_ production. Guan et al. [16] found that atmospheric VOCs in Shijiazhuang were measured at 34.08 ppbv, with alkanes (48.39%) and alkenes (18.93%), as the dominant species. Although alkenes have lower atmospheric concentrations, their chemical reactivity leads them to contribute the most to the OFP of TVOCs, accounting for 47.35%. Moreover, alkenes are the dominant contributor to the Propy-Equiv value of TVOC, accounting for 62.74%. These findings further confirm that reducing alkene emissions is crucial for managing and controlling local O_3_ concentrations.

During photochemical pollution episodes, meteorological conditions act as key modulators of ambient VOC levels and ozone formation. Spearman’s rank correlation of the top 10 measured VOC species shows that relative humidity was significantly positively correlated with 2-benzaldehyde (ρ = 0.90, p < 0.05), while temperature correlated negatively with isopentane (ρ = −0.90, p < 0.05). The generally low wind speeds observed during the 09:00–17:00 sampling window indicate weak atmospheric dilution, which explains the negligible correlation between wind speed and VOC concentrations. These results reflect preliminary observational trends rather than fully validated causal linkages between meteorological drivers and photochemical processes, given the constraint of short-term summer-daytime measurements. Future multi-season, 24-h continuous observations are required to better resolve meteorological controls on local ozone-precursor chemistry.

### Health risk assessment and priority species

The *LCR* values of target VOCs in Qingyang ranged from7.25 × 10^− 11^ to 2.23 × 10^− 6^, all below the tolerable risk level of 1 × 10^− 4^. The urban LCR (9.06 × 10^− 6^) was slightly higher than rural *LCR* (6.75 × 10^− 6^), indicating no prominent carcinogenic risk for local residents. Although these values are below the tolerable risk level (1 × 10^−4^), they exceed the acceptable level (1 × 10^− 6^), indicating that potential long-term carcinogenic concerns cannot be overlooked.

For non-carcinogenic risk, acrolein was identified as the primary risk driver, with HI values of 3.7 in urban areas, 1.1 in rural areas, and 2.9 across the whole study area. The HI of acrolein exceeded the safety threshold of 1 in urban areas but not in rural areas, demonstrating that non-carcinogenic health risks associated with acrolein are a greater concern for the urban population. Consistent with previous findings from a fine‑chemical industrial park in Henan Province [37], acrolein requires priority risk mitigation. In Qingyang, acrolein concentrations were significantly higher in urban zones, particularly in petrochemical‑impacted regions and the city center, which can be attributed to local petrochemical emissions, vehicle exhaust, and photochemical secondary production. Therefore, priority should be given to the risks posed by acrolein in the urban areas, and the control of this substance should be strengthened.

### Limitations

Overall, in Qingyang City, the air quality was poorer in urban areas than in rural areas. Urban air pollution was mainly related to local petrochemical industrial emissions and urban traffic, while the air pollution in rural areas was related to emissions from biomass combustion and diesel-fuel combustion. Given the short sampling campaign (five consecutive days in summer), this work cannot capture weekly, seasonal, or interannual variability in ambient VOCs, constraining the reliability of long-term exposure estimates for lifetime health risk assessment. The 9:00–17:00 sampling window excludes morning rush‑hour peaks and nocturnal VOC buildup, potentially biasing full-day concentration estimates and ozone-precursor assessments. It should also be noted that OFP and propylene-equivalent values in this study rely on literature-derived MIR coefficients for high-NOₓ reference conditions; thus, they should only be interpreted as relative-reactivity ranking metrics rather than absolute predictions of in-situ ozone production. In addition, LCR calculations use uniform exposure parameters without considering factors such as age, occupation, or activity patterns. Combined with short-term sampling, these health-risk results are only preliminary screening-level estimates. The present results characterize VOC signatures under typical summer scenarios and are not intended for annual extrapolation. This study provides a valuable baseline dataset for understanding summer VOC profiles in this under-studied petrochemical city. Future long-term, multi-season monitoring, combined with receptor models (e.g., PMF) and air-quality models, is needed to improve source apportionment accuracy and to identify priority emission sources of high-risk species, which would provide a robust scientific basis for local pollution-control strategies.

## Conclusions

In Qingyang City, the TVOC concentration was higher in the urban areas than in the rural area, and the TVOC composition characteristcs in the urban and rural areas differed. According to the category statistics, the ranking of the volume fractions of the VOCs in the urban areas from high to low was OVOCs, alkanes, HHCs, alkenes, aromatic hydrocarbons, and alkynes. The ranking of the volume fractions of the VOCs in the rural areas from high to low was OVOCs, alkanes, aromatic hydrocarbons, HHCs, alkenes, and alkynes.

Overall, the OVOCs, alkanes, and HHCs were the main components of the local TVOCs. The chemical reactivity of the VOCs was higher in the urban areas than in the rural area. The health risk assessment revealed that the HI was also higher in the urban areas than in the rural area. Acrolein was identified as the priority controlled toxic VOC species with dominant health risk contribution. Controlling the emissions of alkenes and OVOCs can help improve the local air quality.

PCA identified four primary VOC sources: industrial emissions, solvent usage, domestic sources, and industrial/traffic-related halogenated pollutant discharge. This study provides basic observational data for local VOC management. In subsequent long-term monitoring, PMF and air quality models are suggested to further quantify source contributions of high-risk and high-reactivity VOCs, supporting refined atmospheric environmental governance.

## Supporting information

S1 File Supplementary document containing S1-S3 Figs and S1-S4 Tables. (DOCX)

## Abbreviations

VOC: Volatile organic compound
TVOCs: Total volatile organic compounds
HHCs: Halogenated hydrocarbons
SOAs: Secondary organic aerosols
OFP: Ozone formation potential
MIR: Maximum incremental reaction
LCR: Lifetime Carcinogenic Risk
HI: Hazard Index
