# Supplementary material for: Characterization and risk assessment of ambient VOCs in Qingyang city, China
Source: PLoS One. 2026 Sep 28;21(9):e0359213. doi: 10.1371/journal.pone.0359213 (PMC13618953; doi:10.1371/journal.pone.0359213)
Supplement: Supplementary file 1 [file pone.0359213.s001.docx]

Characterization and Risk Assessment of Ambient VOCs in Qingyang City, China

**Weihong Wang^1, 2^,** **Leping He^1, *^, Yongqiang Wei^1^, Fei Dou^1^, Li Ma^1^, Yue Zhang^1^, Yuwei Tie^1^**

^1^Gansu Academy of Eco-environmental Sciences, Gansu 730020, China

^2^Key Laboratory of Industrial Waste Recycling and Regulation of Gansu Province, Gansu 730020, China

*****Correspondence: [gswangweihong@163.com](mailto:gswangweihong@163.com)

**Sampling and VOC analysis**

Sample analysis was performed in the State Key Laboratory of Organic Geochemistry, Guangzhou Institute of Geochemistry, Chinese Academy of Sciences, utilizing a thermal desorption preconcentrator (Entech 7100) coupled with a GC-MS system (Agilent 7890A/5975C). The VOC analysis was performed using a fused-silica capillary column (60 m × 250 µm i.d. × 1.4 µm film thickness) with a stationary phase of 6% cyanopropylphenyl – 94% dimethylpolysiloxane. A 400 mL aliquot of VOC sample was injected onto the third‑stage cold trap of the preconcentrator. In the preconcentration system, the first‑stage cold‑trap capture temperature was set to −150 °C, the capture flow rate was 100 mL/min, and the analytical temperature was 10 °C. The capture temperature of the secondary cold trap was set to −15 °C, the capture flow rate was 10 mL/min, and the desorption temperature was 180°C. The third level focusing temperature was set to −160 °C, and the baking temperature was set to 200 °C. The preconcentrated sample was sent to the gas chromatograph for separation. The starting temperature was set to 35 °C, and after 5 minutes, it was heated to 150°C at a rate of 5 °C/min. After 7 minutes, the temperature was raised to 200 °C at a rate of 10 °C/min, and after 4 minutes, it was detected by a detector. Mass spectrometric detection was operated in full-scan mode with electron-impact (EI) ionization, and the ion-source temperature was maintained at 230°C. Detailed determination procedures followed the national standard HJ 759-2015 [32].

Method detection limits (MDLs) were determined from seven replicate measurements of low‑concentration standard gases. For all target VOC species, MDLs ranged from 0.002 to 0.047 ppbv, which were sufficiently low for ambient air monitoring. Multi‑point calibration curves were prepared using standard gas dilutions. The coefficients of determination (*R*^2^) for all target VOCs were above 0.990, and most were greater than 0.995, indicating good linearity. Relative standard deviations (RSDs) were calculated from replicate analyses. For all VOCs, RSDs ranged from 1% to 7%, indicating satisfactory analytical precision. Laboratory blanks and transport blanks were analyzed for each sample batch. All target compounds in blanks were below their respective MDLs, indicating no significant contamination.

[32] National Environmental Protection Standards of the People's Republic of China (HJ759-2015). Ambient Air—Determination of Volatile Organic Compounds — Collected by Specially-Prepared Canisters and Analyzed by Gas Chromatography/Mass Spectrometry. 2015. [Online].Available: https://www.mee.gov.cn/ywgz/fgbz/bz/bzwb/jcffbz/201510/t20151030_315940.shtml

**S1 Fig.** **Distribution of VOC sample collection locations**

**S2 Fig. Propy-Equiv (ppbv) values and compositions of the different species of alkanes, alkenes, acetylene, aromatics, HHCs, and OVOCs in the (a) urban and (b) rural areas.**

**S3 Fig.** **Non-carcinogenic risks of hazardous VOC species (A–C).**

**S1 Table. Information about VOC species (n=112) measured in this study.**

**S2 Table. The average mixing ratios (mean ± standard deviation; unit: ppbv) of the VOCs observed at the four sites.**

**S3 Table. Principal component analysis of air pollutants total variance explained.**

**S4 Table. Factor loading matrix before and after rotation.**

**S1 Fig. Distribution of VOC sample collection locations**

**S2 Fig.** **Propy-Equiv (ppbv) values and compositions of the different species of alkanes, alkenes, acetylene, aromatics, HHCs, and OVOCs in the (a) urban and (b) rural areas.**

**S3 Fig.** **Non-carcinogenic risks of hazardous VOC species (A–C).**

**S1 Table.** **Information on VOC species (112) measured in this study.**

| **Types** | **No.** | Compounds | **k_OH_ 10^12^** | **MIR** | **Non-carcinogenic** | | **Carcinogenic** | |
| --- | --- | --- | --- | --- | --- | --- | --- | --- |
|  |  |  |  |  | ***RfC*i (μg/m^3^)** | **Source** | ***IUR* (m^3^/μg)** | **Source** |
| Alkanes | 1 | ethane | 0.248 | 0.26 |  |  |  |  |
|  | 2 | propane | 1.09 | 0.46 |  |  |  |  |
|  | 3 | n-butane | 2.36 | 1.08 |  |  |  |  |
|  | 4 | isobutane | 2.12 | 1.17 |  |  |  |  |
|  | 5 | n-pentane | 3.80 | 1.23 | 1000 | RAIS |  |  |
|  | 6 | iso-pentane | 3.60 | 1.36 |  |  |  |  |
|  | 7 | cyclopentane | 4.97 | 2.25 |  |  |  |  |
|  | 8 | 2,2-dimethyl butane | 2.23 | 1.11 |  |  |  |  |
|  | 9 | 2,3-dimethyl butane | 5.78 | 0.91 |  |  |  |  |
|  | 10 | methylcyclopentane | 7.05 | 2.06 |  |  |  |  |
|  | 11 | 2-methyl pentane | 5.20 | 1.41 |  |  |  |  |
|  | 12 | 3-methyl pentane | 5.20 | 1.7 |  |  |  |  |
|  | 13 | n-hexane | 5.20 | 1.15 | 700 | IRIS |  |  |
|  | 14 | cyclohexane | 6.97 | 1.16 | 6000 | IRIS |  |  |
|  | 15 | 2,3-dimethyl pentane | 7.10 | 1.26 |  |  |  |  |
|  | 16 | 2,4-dimethyl pentane | 4.77 | 1.46 |  |  |  |  |
|  | 17 | methylcyclohexane | 9.64 | 1.97 |  |  |  |  |
|  | 18 | 2-methyl hexane | 7.18 | 1.1 |  |  |  |  |
|  | 19 | 3-methyl hexane | 7.18 | 1.51 |  |  |  |  |
|  | 20 | n-heptane | 6.79 | 0.99 | 400 | RAIS |  |  |
|  | 21 | 2,2,4-trimethyl pentane | 3.34 | 1.2 |  |  |  |  |
|  | 22 | 2,3,4-trimethyl pentane | 6.60 | 0.96 |  |  |  |  |
|  | 23 | 2-methyl heptane | 5.20 | 0.99 |  |  |  |  |
|  | 24 | 3-methyl heptane | 8.54 | 1.15 |  |  |  |  |
|  | 25 | n-octane | 8.11 | 0.82 |  |  |  |  |
|  | 26 | n-nonane | 9.70 | 0.71 | 20 | RAIS |  |  |
|  | 27 | n-decane | 11.00 | 0.62 |  |  |  |  |
|  | 28 | n-undecane | 12.30 | 0.55 |  |  |  |  |
|  | 29 | n-dodecane | 13.20 | 0.5 |  |  |  |  |
| Alkenes | 30 | ethene | 8.52 | 8.76 |  |  |  |  |
|  | 31 | propene | 26.30 | 11.37 | 3000 | RAIS |  |  |
|  | 32 | 1-butene | 31.40 | 9.42 |  |  |  |  |
|  | 33 | cis-2-butene | 56.40 | 13.89 |  |  |  |  |
|  | 34 | trans-2-butene | 64.00 | 14.79 |  |  |  |  |
|  | 35 | 1,3-butadiene | 66.60 | 13.47 | 2 | IRIS | 0.00003 | IRIS |
|  | 36 | 1-pentene | 31.40 | 6.97 |  |  |  |  |
|  | 37 | Isoprene | 100.00 | 10.28 |  |  |  |  |
|  | 38 | cis-2-pentene | 65.00 | 10.07 |  |  |  |  |
|  | 39 | trans-2-pentene | 67.00 | 10.25 |  |  |  |  |
|  | 40 | 1-hexene | 37.00 | 5.28 |  |  |  |  |
| Alkynes | 41 | acetylene | 0.90 | 0.93 |  |  |  |  |
| Aromatics | 42 | benzene | 1.22 | 0.69 | 30 | IRIS | 0.0000078 | IRIS |
|  | 43 | toluene | 5.63 | 3.88 | 5000 | IRIS |  |  |
|  | 44 | m/p-xylene | 23.10 | 15.21 | 100 | IRIS |  |  |
|  | 45 | o-xylene | 13.60 | 7.44 | 100 | IRIS |  |  |
|  | 46 | ethyl benzene | 7.00 | 2.93 | 1000 | IRIS | 0.0000025 | RAIS |
|  | 47 | styrene | 58.00 | 1.65 | 1000 | IRIS |  |  |
|  | 48 | m-ethyl toluene | 18.60 | 7.21 |  |  |  |  |
|  | 49 | p-ethyl toluene | 11.80 | 4.32 |  |  |  |  |
|  | 50 | o-ethyl toluene | 11.90 | 5.43 |  |  |  |  |
|  | 51 | 1,2,3-trimethyl benzene | 32.70 | 11.66 | 60 | IRIS |  |  |
|  | 52 | 1,2,4-trimethyl benzene | 32.50 | 8.64 | 60 | IRIS |  |  |
|  | 53 | 1,3,5-trimethyl benzene | 56.70 | 11.44 | 60 | IRIS |  |  |
|  | 54 | n-propyl benzene | 5.80 | 1.95 | 1000 | RAIS |  |  |
|  | 55 | isopropyl benzene | 6.30 | 2.43 | 400 | IRIS |  |  |
|  | 56 | m-diethyl benzene | 29.00 | 6.92 |  |  |  |  |
|  | 57 | p-diethyl benzene | 29.00 | 4.31 |  |  |  |  |
| HHCs | 58 | methyl chloride | 0.00836 | 0.036 | 90 | IRIS |  |  |
|  | 59 | dichloromethane |  | 0.039 | 600 | IRIS | 0.00000001 | IRIS |
|  | 60 | chloroform |  | 0.02 | 98 | [a] | 0.000023 | IRIS |
|  | 61 | methyl bromide |  | 0.018 | 5 |  |  |  |
|  | 62 | dichlorodifluoromethane |  |  | 100 | [a] |  |  |
|  | 63 | trichlorofluoromethane |  |  |  |  |  |  |
|  | 64 | bromodichloromethane |  |  |  |  | 0.000037 | [a] |
|  | 65 | dibronochloromethane |  |  |  |  |  |  |
|  | 66 | tribromomethane |  |  |  |  | 0.0000011 | [a] |
|  | 67 | ethyl chloride |  | 0.27 | 10000 | IRIS |  |  |
|  | 68 | 1,1-dichloroethane |  | 0.065 | 500 | [a] | 0.0000016 | [a] |
|  | 69 | 1,2-dichloroethane |  | 0.21 | 7 | RAIS | 0.000026 | RAIS |
|  | 70 | 1,1,1-trichloroethane |  | 0.005 | 7000 |  |  |  |
|  | 71 | 1,1,2-trichloroethane |  | 0.082 | 0.2 | RAIS | 0.000016 | IRIS |
|  | 72 | 1,1,2,2-tetrachloroethane |  |  |  |  | 0.000058 | [a] |
|  | 73 | 1,1,2,2-tetrafluoro-1,2-dichloroethane |  |  |  |  |  |  |
|  | 74 | 1,1,2-trichloro-1,2,2-trifluoroethane |  |  |  |  |  |  |
|  | 75 | 1,2-dibromoethane |  |  | 9 | IRIS | 0.0006 | IRIS |
|  | 76 | 1,2-dichloropropane |  | 0.28 | 4 | IRIS |  |  |
|  | 77 | vinyl chloride |  | 2.7 | 100 | IRIS | 0.0000088 | IRIS |
|  | 78 | 1,1-dichloroethene |  | 1.69 | 200 | IRIS |  |  |
|  | 79 | cis-1,2-dichloroethene |  | 1.65 |  |  |  |  |
|  | 80 | trans-1,2-dichloroethene |  | 1.65 | 40 | RAIS |  |  |
|  | 81 | trichloroethylene |  | 0.61 | 2 | [b] | 0.0000041 | [b] |
|  | 82 | perchloroethylene |  | 0.029 | 40 |  | 0.00000026 |  |
|  | 83 | cis-1,3-dichloropropene |  | 3.61 |  |  |  |  |
|  | 84 | trans-1,3-dichloropropene |  | 4.92 | 20 | [a] | 0.000004 | [a] |
|  | 85 | hexachloro-1,3-butadiene |  |  | 90 | [a] | 0.000022 | IRIS |
|  | 86 | monochlorobenzene |  | 0.31 | 50 | RAIS |  |  |
|  | 87 | o-dichlorobenzene |  | 0.171 | 200 | RAIS |  |  |
|  | 88 | m-dichlorobenzene |  |  |  |  |  |  |
|  | 89 | p-dichlorobenzene |  | 0.171 | 800 | IRIS |  |  |
|  | 90 | 1,2,4-trichlorobenzene |  |  | 200 | [a] |  |  |
|  | 91 | benzyl chloride |  |  | 1 | RAIS | 0.000049 | RAIS |
| OVOCs | 92 | isopropyl alcohol |  | 0.59 | 200 | RAIS |  |  |
|  | 93 | methyl t-butyl ether | 2.83 | 0.7 | 3000 | IRIS | 0.00000026 | RAIS |
|  | 94 | acetaldehyde | 15.00 | 6.34 | 9 | IRIS | 0.0000022 | IRIS |
|  | 95 | propionaldehyde | 20.00 | 6.83 | 8 | IRIS |  |  |
|  | 96 | acrolein | 19.90 | 7.24 | 0.02 | IRIS |  |  |
|  | 97 | crotonaldehyde |  | 9.14 |  |  |  |  |
|  | 98 | methacrolein | 29.00 | 5.84 |  |  |  |  |
|  | 99 | butanal | 24.00 | 5.75 |  |  |  |  |
|  | 100 | pentanal | 28.00 | 4.89 |  |  |  |  |
|  | 101 | hexanal | 30.00 | 4.18 |  |  |  |  |
|  | 102 | m-tolualdehyde | 17 |  |  |  |  |  |
|  | 103 | benzaldehyde | 12.00 | −0.67 |  |  |  |  |
|  | 104 | acetone | 0.17 | 0.35 | 31000 | [a] |  |  |
|  | 105 | methyl ethyl ketone | 1.22 | 1.43 | 5000 | IRIS |  |  |
|  | 106 | 4-methyl-2-pentanone | 13.00 | 3.74 | 3000 | IRIS |  |  |
|  | 107 | methyl n-butyl ketone | 9.10 | 3 | 30 | IRIS |  |  |
|  | 108 | vinyl acetate |  | 3.11 | 200 | IRIS |  |  |
|  | 109 | ethyl acetate |  | 0.59 | 70 | RAIS |  |  |
|  | 110 | methyl methacrylate |  | 15.22 | 700 | IRIS |  |  |
|  | 111 | tetrahydrofuran |  | 4.1 | 2000 | IRIS |  |  |
|  | 112 | 1,4-dioxane |  | 2.48 | 30 | IRIS | 0.000005 | IRIS |

IRIS: the Integrated Risk Information System, https://www.epa.gov/iris.

or <https://iris.epa.gov/AdvancedSearch/?keyword=vocs>

RAIS: the Risk Assessment Information System, <https://rais.ornl.gov/index.html>.

ATSDR: Agency for Toxic Substances and Disease Registry, <https://www.atsdr.cdc.gov/>.

[a]: Han M.J., Ren G., Zhao X.N., Zhang X.D., Lin H., Liu D., Wang L. Spatial heterogeneity of volatile organic compound pollution in a typical industrial park based on multi-point online monitoring: Pollution characteristics, health risks, and priority-controlled species. Atmospheric Environment. 2024. 338,120852. <https://doi.org/10.1016/j.atmosenv.2024.120852>.

[b]: Liu Y.F., Kong L.W., Liu X.N., Zhang Y.P., Li C.L., Zhang Y.Y., Zhang C., Qu Y., An J.L., Ma D.P., Tan Q.W., Feng M., Zha S.P. Characteristics, secondary transformation, and health risk assessment of ambient volatile organic compounds (VOCs) in urban Beijing, China. Atmospheric Pollution Research. 2021.12, 33–46. <https://doi.org/10.1016/j.apr.2021.01.013>.

**S2 Table.** **The average mixing ratios (mean ± standard deviation; unit: ppbv) of the VOCs observed at the four sites.**

| **Species** | **No.** | VOCs | **1** | **2** | **3** | **4** | **Average** |
| --- | --- | --- | --- | --- | --- | --- | --- |
| Alkanes | 1 | ethane | 0.79±0.21 | 0.73±0.43 | 0.46±0.08 | 1.20±0.91 | 0.81±0.55 |
|  | 2 | propane | 0.53±0.03 | 0.51±0.12 | 0.45±0.07 | 0.51±0.13 | 0.50±0.09 |
|  | 3 | n-butane | 0.66±0.05 | 1.23±0.83 | 3.14±0.26 | 0.05±0.05 | 1.31±1.28 |
|  | 4 | isobutane | 1.26±0.44 | 0.81±0.58 | 1.49±0.31 | 0.00±0.00 | 0.87±0.70 |
|  | 5 | n-pentane | 0.63±0.12 | 0.38±0.22 | 0.90±0.19 | 0.04±0.03 | 0.48±0.36 |
|  | 6 | iso-pentane | 1.01±0.79 | 0.88±0.80 | 2.92±0.63 | 0.19±0.17 | 1.26±1.22 |
|  | 7 | cyclopentane | 1.85±0.00 | 0.18±0.16 | 0.23±0.27 | 0.02±0.01 | 0.43±0.68 |
|  | 8 | 2,2-dimethyl butane | 1.10±0.00 | 0.20±0.19 | 0.10±0.11 | 0.00±0.00 | 0.26±0.40 |
|  | 9 | 2,3-dimethyl butane | 3.37±0.00 | 0.66±0.64 | 0.42±0.38 | 0.00±0.00 | 0.87±1.24 |
|  | 10 | methylcyclopentane | 0.53±0.39 | 0.51±0.28 | 0.49±0.15 | 1.15±1.24 | 0.67±0.68 |
|  | 11 | 2-methyl pentane | 0.37±0.30 | 0.28±0.16 | 1.58±1.45 | 0.04±0.03 | 0.58±0.94 |
|  | 12 | 3-methyl pentane | 0.16±0.00 | 0.20±0.11 | 0.99±0.92 | 0.02±0.02 | 0.36±0.61 |
|  | 13 | n-hexane | 0.83±0.71 | 1.64±1.85 | 0.52±0.25 | 0.31±0.14 | 0.82±1.06 |
|  | 14 | cyclohexane | 0.14±0.08 | 0.23±0.15 | 0.12±0.03 | 0.39±0.21 | 0.22±0.17 |
|  | 15 | 2,3-dimethyl pentane | 0.07±0.06 | 0.18±0.22 | 0.08±0.04 | 0.21±0.11 | 0.14±0.13 |
|  | 16 | 2,4-dimethyl pentane | 0.09±0.04 | 0.09±0.05 | 0.10±0.02 | 0.26±0.15 | 0.14±0.10 |
|  | 17 | methylcyclohexane | 0.13±0.13 | 0.34±0.42 | 0.19±0.12 | 0.27±0.15 | 0.23±0.23 |
|  | 18 | 2-methyl hexane | 0.13±0.10 | 0.54±0.69 | 0.29±0.17 | 0.46±0.30 | 0.36±0.39 |
|  | 19 | 3-methyl hexane | 0.14±0.12 | 0.61±0.79 | 0.27±0.13 | 0.45±0.26 | 0.37±0.43 |
|  | 20 | n-heptane | 0.11±0.10 | 0.46±0.59 | 0.13±0.08 | 0.57±0.56 | 0.32±0.43 |
|  | 21 | 2,2,4-trimethyl pentane | 0.04±0.02 | 0.03±0.01 | 0.17±0.09 | 1.49±1.92 | 0.43±1.08 |
|  | 22 | 2,3,4-trimethyl pentane | 0.05±0.03 | 0.16±0.19 | 0.10±0.05 | 0.10±0.06 | 0.11±0.10 |
|  | 23 | 2-methyl heptane | 0.06±0.04 | 0.24±0.28 | 0.09±0.04 | 0.12±0.07 | 0.13±0.15 |
|  | 24 | 3-methyl heptane | 0.05±0.03 | 0.17±0.20 | 0.09±0.03 | 0.11±0.06 | 0.10±0.11 |
|  | 25 | n-octane | 0.15±0.15 | 0.69±0.91 | 0.14±0.07 | 0.93±1.16 | 0.48±0.77 |
|  | 26 | n-nonane | 0.08±0.07 | 0.28±0.35 | 0.11±0.06 | 0.25±0.14 | 0.18±0.20 |
|  | 27 | n-decane | 0.10±0.05 | 0.05±0.03 | 0.26±0.13 | 0.15±0.07 | 0.14±0.11 |
|  | 28 | n-undecane | 0.05±0.01 | 0.10±0.08 | 0.56±0.43 | 0.63±0.33 | 0.33±0.37 |
|  | 29 | n-dodecane | 0.00±0.00 | 0.19±0.17 | 1.29±1.05 | 1.20±1.18 | 0.64±0.92 |
| Alkenes | 30 | ethene | 0.59±0.22 | 0.48±0.41 | 0.37±0.07 | 1.40±1.68 | 0.71±0.90 |
|  | 31 | propene | 0.05±0.03 | 0.07±0.06 | 0.02±0.02 | 0.06±0.05 | 0.05±0.04 |
|  | 32 | 1-butene | 0.61±0.55 | 1.01±0.57 | 2.78±1.90 | 0.01±0.01 | 1.10±1.42 |
|  | 33 | cis-2-butene | 0.14±0.08 | 0.62±0.55 | 1.14±1.32 | 0.00±0.00 | 0.48±0.80 |
|  | 34 | trans-2-butene | 0.14±0.08 | 0.66±0.76 | 1.83±2.33 | 0.02±0.02 | 0.66±1.34 |
|  | 35 | 1,3-butadiene | 1.01±1.02 | 0.66±0.77 | 0.27±0.23 | 0.02±0.02 | 0.49±0.71 |
|  | 36 | 1-pentene | 0.91±0.71 | 0.35±0.35 | 0.21±0.19 | 0.00±0.00 | 0.37±0.51 |
|  | 37 | isoprene | 0.22±0.21 | 0.35±0.18 | 0.89±0.71 | 0.02±0.02 | 0.37±0.48 |
|  | 38 | cis-2-pentene | 0.44±0.41 | 0.19±0.19 | 0.25±0.27 | 0.01±0.01 | 0.22±0.29 |
|  | 39 | trans-2-pentene | 0.88±0.90 | 0.36±0.32 | 0.60±0.64 | 0.02±0.02 | 0.46±0.62 |
|  | 40 | 1-hexene | 0.57±0.52 | 0.14±0.13 | 0.08±0.07 | 0.00±0.00 | 0.20±0.33 |
| Alkynes | 41 | Acetylene | 0.44±0.24 | 0.54±0.30 | 0.34±0.04 | 0.33±0.09 | 0.41±0.20 |
| Aromatics | 42 | Benzene | 1.49±1.05 | 1.24±0.76 | 1.30±0.49 | 1.64±1.58 | 1.42±0.98 |
|  | 43 | Toluene | 1.26±1.16 | 0.18±0.10 | 1.41±0.06 | 1.46±0.98 | 1.08±0.88 |
|  | 44 | m/p-xylene | 0.08±0.08 | 0.08±0.07 | 0.49±0.31 | 1.21±0.97 | 0.47±0.67 |
|  | 45 | o-xylene | 0.05±0.03 | 0.04±0.03 | 0.40±0.24 | 0.79±0.61 | 0.32±0.44 |
|  | 46 | ethyl benzene | 0.03±0.02 | 0.04±0.04 | 0.46±0.36 | 1.42±1.21 | 0.49±0.82 |
|  | 47 | Styrene | 0.29±0.26 | 0.08±0.07 | 0.07±0.06 | 0.79±0.80 | 0.31±0.49 |
|  | 48 | m-ethyl toluene | 0.11±0.05 | 0.18±0.17 | 0.15±0.05 | 0.41±0.24 | 0.21±0.18 |
|  | 49 | p-ethyl toluene | 0.07±0.02 | 0.11±0.12 | 0.10±0.04 | 0.28±0.16 | 0.14±0.13 |
|  | 50 | o-ethyl toluene | 0.54±0.55 | 0.11±0.04 | 0.34±0.24 | 0.39±0.39 | 0.34±0.37 |
|  | 51 | 1,2,3-trimethyl benzene | 0.11±0.03 | 0.10±0.04 | 0.20±0.12 | 0.30±0.27 | 0.18±0.16 |
|  | 52 | 1,2,4-trimethyl benzene | 0.20±0.06 | 0.13±0.02 | 0.22±0.09 | 0.32±0.26 | 0.22±0.15 |
|  | 53 | 1,3,5-trimethyl benzene | 0.14±0.07 | 0.09±0.03 | 0.27±0.16 | 0.47±0.53 | 0.24±0.29 |
|  | 54 | n-propyl benzene | 0.10±0.10 | 0.25±0.23 | 0.13±0.12 | 0.35±0.21 | 0.21±0.19 |
|  | 55 | isopropyl benzene | 0.03±0.02 | 0.01±0.01 | 0.05±0.04 | 0.19±0.20 | 0.07±0.12 |
|  | 56 | m-diethyl benzene | 0.05±0.04 | 0.03±0.02 | 0.16±0.13 | 0.37±0.34 | 0.15±0.22 |
|  | 57 | p-diethyl benzene | 0.07±0.01 | 0.07±0.01 | 0.07±0.01 | 0.08±0.02 | 0.07±0.01 |
| HHCs | 58 | methyl chloride | 0.69±0.69 | 2.95±2.11 | 3.32±0.69 | 0.03±0.03 | 1.75±1.80 |
|  | 59 | Dichloromethane | 0.58±0.49 | 0.95±0.55 | 3.00±2.34 | 0.12±0.11 | 1.16±1.60 |
|  | 60 | Chloroform | 0.57±0.53 | 1.08±0.88 | 0.24±0.04 | 0.21±0.06 | 0.52±0.59 |
|  | 61 | methyl bromide | 0.35±0.37 | 0.02±0.02 | 0.07±0.05 | 0.00±0.00 | 0.11±0.22 |
|  | 62 | dichlorodifluoromethane | 0.00±0.00 | 0.00±0.00 | 0.00±0.00 | 0.00±0.00 | 0.00±0.00 |
|  | 63 | trichlorofluoromethane | 0.31±0.32 | 0.75±0.50 | 0.95±0.65 | 0.01±0.01 | 0.50±0.55 |
|  | 64 | bromodichloromethane | 0.03±0.01 | 0.15±0.18 | 0.03±0.01 | 0.13±0.12 | 0.09±0.11 |
|  | 65 | dibromochloromethane | 0.15±0.15 | 0.10±0.09 | 0.18±0.15 | 0.09±0.06 | 0.13±0.12 |
|  | 66 | tribromomethane | 0.02±0.00 | 0.15±0.11 | 0.03±0.00 | 0.03±0.00 | 0.05±0.07 |
|  | 67 | ethyl chloride | 0.49±0.51 | 0.17±0.18 | 0.15±0.07 | 0.00±0.00 | 0.20±0.31 |
|  | 68 | 1,1-dichloroethane | 0.12±0.12 | 0.25±0.23 | 0.31±0.27 | 0.00±0.00 | 0.17±0.21 |
|  | 69 | 1,2-dichloroethane | 1.11±0.87 | 0.86±0.93 | 0.81±0.26 | 0.37±0.25 | 0.79±0.66 |
|  | 70 | 1,1,1-trichloroethane | 0.03±0.00 | 0.03±0.00 | 0.03±0.00 | 0.04±0.00 | 0.03±0.00 |
|  | 71 | 1,1,2-trichloroethane | 0.18±0.21 | 0.25±0.31 | 0.05±0.01 | 0.07±0.03 | 0.14±0.19 |
|  | 72 | 1,1,2,2-Tetrachloroethane | 0.02±0.00 | 0.18±0.22 | 0.03±0.02 | 0.03±0.01 | 0.07±0.12 |
|  | 73 | 1,1,2,2-Tetrafluoro-1,2-Dichloroethane | 0.43±0.39 | 0.06±0.03 | 0.05±0.03 | 0.00±0.00 | 0.13±0.25 |
|  | 74 | 1,1,2-Trichloro-1,2,2-Trifluoroethane | 0.11±0.10 | 0.20±0.12 | 0.20±0.13 | 0.00±0.00 | 0.13±0.13 |
|  | 75 | 1,2-dibromoethane | 0.03±0.00 | 0.03±0.00 | 0.03±0.00 | 0.03±0.00 | 0.03±0.00 |
|  | 76 | 1,2-dichloropropane | 0.46±0.29 | 0.28±0.16 | 1.37±0.52 | 1.85±1.09 | 0.99±0.88 |
|  | 77 | vinyl chloride | 0.62±0.64 | 0.28±0.18 | 0.26±0.22 | 0.00±0.00 | 0.29±0.39 |
|  | 78 | 1,1-dichloroethene | 0.21±0.19 | 0.19±0.19 | 0.05±0.07 | 0.00±0.00 | 0.11±0.15 |
|  | 79 | cis-1,2-dichloroethene | 0.08±0.07 | 0.30±0.38 | 0.03±0.00 | 0.02±0.01 | 0.11±0.21 |
|  | 80 | trans-1,2-dichloroethene | 0.46±0.44 | 0.52±0.63 | 0.19±0.13 | 0.04±0.04 | 0.30±0.41 |
|  | 81 | Trichloroethylene | 0.04±0.01 | 0.30±0.38 | 0.07±0.03 | 0.06±0.02 | 0.12±0.21 |
|  | 82 | Perchloroethylene | 0.26±0.29 | 0.05±0.03 | 0.31±0.29 | 0.18±0.12 | 0.20±0.22 |
|  | 83 | cis-1,3-dichloropropene | 0.06±0.01 | 0.41±0.49 | 0.06±0.01 | 0.06±0.01 | 0.15±0.27 |
|  | 84 | trans-1,3-dichloropropene | 0.10±0.02 | 0.86±0.63 | 0.13±0.03 | 0.14±0.05 | 0.28±0.40 |
|  | 85 | hexachloro-1,3-butadiene | 0.03±0.00 | 0.03±0.01 | 0.05±0.03 | 0.03±0.00 | 0.03±0.02 |
|  | 86 | Monochlorobenzene | 0.05±0.02 | 0.62±0.82 | 0.10±0.05 | 0.09±0.05 | 0.22±0.45 |
|  | 87 | o-dichlorobenzene | 0.14±0.02 | 0.26±0.14 | 0.15±0.02 | 0.25±0.09 | 0.20±0.10 |
|  | 88 | m-dichlorobenzene | 0.04±0.02 | 0.17±0.17 | 0.04±0.02 | 0.10±0.09 | 0.09±0.11 |
|  | 89 | p-dichlorobenzene | 0.05±0.02 | 0.17±0.15 | 0.05±0.02 | 0.11±0.08 | 0.10±0.10 |
|  | 90 | 1,2,4-trichlorobenzene | 0.04±0.02 | 0.43±0.32 | 0.03±0.00 | 0.21±0.21 | 0.16±0.23 |
|  | 91 | benzyl chloride | 0.18±0.01 | 0.18±0.02 | 0.22±0.05 | 0.25±0.10 | 0.21±0.06 |
| OVOCs | 92 | isopropyl alcohol | 0.39±0.32 | 0.62±0.28 | 0.85±0.08 | 0.09±0.08 | 0.49±0.35 |
|  | 93 | methyl t-butyl ether | 0.97±0.99 | 1.13±1.40 | 1.32±1.43 | 0.03±0.03 | 0.86±1.15 |
|  | 94 | Acetaldehyde | 0.95±0.54 | 1.40±0.10 | 1.59±0.11 | 3.83±3.50 | 1.97±2.04 |
|  | 95 | Propionaldehyde | 0.24±0.14 | 0.65±0.30 | 2.35±0.82 | 0.21±0.19 | 0.86±0.99 |
|  | 96 | Acrolein | 0.34±0.24 | 0.79±0.36 | 0.78±0.21 | 0.14±0.12 | 0.51±0.37 |
|  | 97 | Crotonaldehyde | 0.90±0.53 | 1.56±0.99 | 1.76±0.62 | 0.24±0.15 | 1.12±0.85 |
|  | 98 | Methacrolein | 2.87±2.98 | 1.10±1.34 | 0.72±0.39 | 0.03±0.02 | 1.18±1.86 |
|  | 99 | Butanal | 0.24±0.19 | 1.94±2.43 | 0.99±0.59 | 0.07±0.06 | 0.81±1.38 |
|  | 100 | Pentanal | 0.69±0.63 | 0.28±0.14 | 1.04±0.33 | 0.47±0.23 | 0.62±0.45 |
|  | 101 | Hexanal | 0.38±0.19 | 0.20±0.12 | 0.48±0.53 | 0.08±0.01 | 0.29±0.31 |
|  | 102 | m-tolualdehyde | 0.43±0.25 | 0.21±0.23 | 0.15±0.11 | 0.53±0.64 | 0.33±0.37 |
|  | 103 | Benzaldehyde | 0.25±0.11 | 0.90±0.97 | 0.78±0.44 | 2.21±1.25 | 1.04±1.06 |
|  | 104 | Acetone | 0.95±0.55 | 0.88±0.28 | 0.77±0.05 | 0.84±0.77 | 0.86±0.46 |
|  | 105 | methyl ethyl ketone | 0.20±0.20 | 0.09±0.07 | 0.61±0.39 | 0.01±0.01 | 0.23±0.31 |
|  | 106 | 4-methyl-2-pentanone | 0.08±0.05 | 0.24±0.24 | 0.76±0.27 | 0.43±0.25 | 0.38±0.33 |
|  | 107 | methyl n-butyl ketone | 0.08±0.02 | 0.07±0.04 | 0.10±0.03 | 0.32±0.28 | 0.15±0.17 |
|  | 108 | vinyl acetate | 0.19±0.16 | 0.29±0.16 | 1.53±1.34 | 0.04±0.04 | 0.52±0.87 |
|  | 109 | ethyl acetate | 0.68±0.38 | 0.41±0.31 | 1.11±0.29 | 0.12±0.03 | 0.58±0.46 |
|  | 110 | methyl methacrylate | 0.07±0.03 | 0.73±0.96 | 0.20±0.16 | 0.26±0.16 | 0.31±0.52 |
|  | 111 | Tetrahydrofuran | 1.01±1.31 | 0.36±0.42 | 0.22±0.22 | 1.74±1.82 | 0.83±1.22 |
|  | 112 | 1,4-dioxane | 0.13±0.06 | 0.08±0.05 | 0.16±0.09 | 0.29±0.25 | 0.16±0.15 |

**S3 Table. Principal component analysis of air pollutants total variance explained.**

| Component | Initial Eigenvalues | | | Extraction Sums of Squared Loadings | | | Rotation Sums of Squared Loadings | | |
| --- | --- | --- | --- | --- | --- | --- | --- | --- | --- |
|  | Total | % of Variance | Cumulative % | Total | % of Variance | Cumulative % | Total | % of Variance | Cumulative % |
| PC1 | 46.40 | 38.00 | 38.00 | 46.401 | 38.00 | 38.00 | 42.16 | 34.52 | 34.52 |
| PC2 | 30.03 | 24.59 | 62.59 | 30.029 | 24.59 | 62.59 | 28.74 | 23.57 | 58.09 |
| PC3 | 22.42 | 18.36 | 80.95 | 22.423 | 18.36 | 80.95 | 24.89 | 20.38 | 78.47 |
| PC4 | 13.59 | 11.13 | 92.08 | 13.586 | 11.13 | 92.08 | 16.66 | 13.61 | 92.08 |
| PC5 | 5.82 | 4.77 | 96.85 | — | — | — | — | — | — |
| PC6 | 2.32 | 1.90 | 98.75 | — | — | — | — | — | — |
| PC7 | 0.78 | 0.63 | 99.38 | — | — | — | — | — | — |
| PC8 | 0.32 | 0.26 | 99.64 | — | — | — | — | — | — |
| PC9 | 0.26 | 0.21 | 99.85 | — | — | — | — | — | — |
| PC10 | 0.18 | 0.14 | 100.00 | — | — | — | — | — | — |

**S4 Table.** **Factor loading matrix before and after rotation.**

| VOC_s_ | Factor loading matrix before rotation | | | | Factor loading matrix after orthogonal rotation of maximum variance | | | |
| --- | --- | --- | --- | --- | --- | --- | --- | --- |
|  | PC1 | PC2 | PC3 | PC4 | PC1 | PC2 | PC3 | PC4 |
| Tetrahydrofuran | 0.034 | -0.158 | -0.040 | 0.143 | 0.089 | -0.187 | 0.012 | 0.112 |
| Methyl methacrylate | -0.026 | -0.159 | -0.049 | 0.143 | 0.045 | -0.192 | 0.009 | 0.120 |
| m-Ethyltoluene | -0.065 | -0.076 | 0.096 | 0.139 | -0.022 | -0.054 | 0.125 | 0.157 |
| Ethyl acetate | 0.034 | -0.158 | -0.040 | 0.143 | 0.089 | -0.187 | 0.012 | 0.112 |
| Benzaldehyde | -0.047 | -0.100 | 0.167 | -0.057 | -0.066 | -0.042 | 0.199 | -0.024 |
| o-Dichlorobenzene | 0.085 | -0.149 | 0.066 | -0.078 | 0.113 | -0.102 | 0.088 | -0.066 |
| Chlorobenzene | 0.072 | -0.090 | 0.159 | -0.066 | 0.099 | -0.041 | 0.185 | -0.033 |
| 1,2-Dichloropropane | 0.129 | -0.065 | -0.052 | 0.079 | 0.157 | -0.012 | -0.032 | 0.090 |
| cis-1,3-Dichloropropene | 0.066 | 0.146 | 0.075 | -0.090 | 0.042 | 0.166 | 0.099 | -0.054 |
| trans-1,3-Dichloropropene | 0.059 | 0.065 | -0.152 | -0.079 | 0.022 | 0.088 | -0.187 | -0.041 |

Extraction Method: Principal Component Analysis.

Rotation Method: Varimax with Kaiser Normalization.
